# Global existence of Dirac-wave maps with curvature term on expanding spacetimes

**DOI:** 10.1007/s00526-018-1389-8

**Published:** 2018-07-20

**Authors:** Volker Branding, Klaus Kröncke

**Affiliations:** 1 Faculty of Mathematics, University of Vienna, Oskar-Morgenstern-Platz 1, 1090 Vienna, Austria; 2 Department of Mathematics, University of Hamburg, Bundesstrasse 55, 20146 Hamburg, Germany

**Keywords:** 58J45, 53C27, 53C50, 35L71

## Abstract

We prove the global existence of Dirac-wave maps with curvature term with small initial data on globally hyperbolic manifolds of arbitrary dimension which satisfy a suitable growth condition. In addition, we also prove a global existence result for wave maps under similar assumptions.

## Introduction and results

Wave maps are among the fundamental variational problems in differential geometry. They are defined as critical points of the Dirichlet energy for a map between two manifolds, where one assumes that the domain manifold is Lorentzian and the target manifold is Riemannian. More precisely, let (*M*, *h*) be a globally hyperbolic Lorentzian manifold, (*P*, *G*) a Riemannian manifold and $\phi :M\rightarrow P$ a map. Squaring the norm of its differential gives rise to the Dirichlet energy, whose critical points are given by the *wave map equation*, which is $\tau (\phi )=0$, where $\tau (\phi ):=-h^{\alpha \beta }\nabla _{\partial _\alpha }d\phi (\partial _\beta )$.

The wave map equation is a second-order semilinear hyperbolic system, that is the natural analogue of the harmonic map equation for maps between Riemannian manifolds. Wave maps are also well-studied in the physics literature, they appear as critical points of the *Polyakov action* in bosonic string theory for a string with Lorentzian worldsheet.

There are many articles that study wave maps in the case that the domain is Minkowski space. We cannot give an exhaustive list of the results here, but want to mention the influential works of Klainerman [15], Tataru [26], Klainerman and Selberg [17], Klainerman and Machedon [16], Tao [24, 25] and Shatah and Struwe [22, 23].

There are less articles that consider the case of a domain being a non-flat globally hyperbolic manifold. Here, we want to mention the works of Choquet-Bruhat [9, 10] for wave maps on Robertson–Walker spacetimes and several recent articles that consider wave maps on non-flat backgrounds [11, 14, 18, 20]. To obtain an overview on the current status of research on the wave map equation we refer to the recent book [12].

In modern quantum field theory one considers extensions of the wave map system, one of them being the supersymmetric nonlinear $\sigma$-model [1]. Recently, there has been a lot of interested in this model from a mathematical perspective. In mathematical terms, the model leads to a geometric variational problem that couples a map between manifolds to spinor fields.

Most of the mathematical research on this model so far is concerned with the case that both manifolds are Riemannian leading to the notion of Dirac-harmonic maps [8] and Dirac-harmonic map with curvature term [4, 7], which represent semilinear elliptic problems. At present, many results regarding the geometric and analytic structure of Dirac-harmonic maps and Dirac-harmonic maps with curvature are known [5], but no existence result for these kind of equations could be achieved.

In case that the domain manifold has a Lorentzian metric the critical points of the supersymmetric nonlinear $\sigma$-model lead to a system of the wave map equation coupled to spinor fields. For this system two existence results are available [6, 13] for the domain being two-dimensional Minkowski space.

In order to couple the wave map equation to spinor fields we have to recall some concepts from spin geometry on globally hyperbolic manifolds. We have to make the additional assumption that the manifold $M$ is spin, which guarantees the existence of the spinor bundle $SM$. Sections in the spinor bundle are called spinors. Moreover, we fix a spin structure. The spinor bundle is a vector bundle on which we choose a metric connection compatible with the hermitian scalar product denoted by $\langle \cdot ,\cdot \rangle _{S M}$. On the spinor bundle we have the Clifford multiplication of spinors with tangent vectors, which satisfies

$$\begin{aligned} \langle \psi ,X\cdot \xi \rangle _{SM}=\langle X\cdot \psi ,\xi \rangle _{SM} \end{aligned}$$

for all $X\in TM,\psi ,\xi \in \Gamma (SM)$. In addition, the Clifford relations

$$\begin{aligned} X\cdot Y+Y\cdot X =-2h(X,Y) \end{aligned}$$

hold for all $X,Y\in TM$, where $h$ represents the metric on $M$.

The natural operator acting on spinors is the *Dirac operator*, which is defined as the composition of applying the covariant derivative first and Clifford multiplication in the second step. More precisely, the Dirac operator acting on spinors is given by

$$\begin{aligned} \not \partial :=h^{\alpha \beta }\partial _\alpha \cdot \nabla _{\partial _\beta }. \end{aligned}$$

The Dirac operator is a linear first order hyperbolic differential operator. For more background on spin geometry on globally hyperbolic manifolds we refer to [2, 3]. The Dirac operator itself is anti-self-adjoint with respect to the $L^2$-norm. However, the combination $i\not \partial$ yields a self-adjoint operator, that is

$$\begin{aligned} \int _M\langle i\not \partial \xi ,\psi \rangle \text { }dV_h=\int _M\langle \xi ,i\not \partial \psi \rangle \text { }dV_h. \end{aligned}$$

The square of the Dirac operator satisfies the *Schrödinger–Lichnerowicz* formula

$$\begin{aligned} \not \partial ^2=\Box +\frac{\mathrm {scal}^M}{4}, \end{aligned}$$

where $\mathrm {scal}^M$ denotes the scalar curvature of the manifold $M$. Note that $\not \partial ^2$ is a wave-type operator.

In quantum field theory one usually considers spinors that are twisted by some additional vector bundle. Here, we consider the case that the spinor bundle is twisted with the pullback of the tangent bundle from the target manifold. More precisely, we are considering the bundle $SM\otimes \phi ^*TP$ and sections in this bundle are called *vector spinors*. We obtain a connection on $SM\otimes \phi ^*TP$ by setting

$$\begin{aligned} \nabla ^{SM\otimes \phi ^*TP}=\nabla ^{SM}\otimes \mathbb {1}^{\phi ^*TP}+\mathbb {1}^{SM}\otimes \nabla ^{\phi ^*TP}. \end{aligned}$$

Note that Clifford multiplication on the twisted bundle is defined by acting only on the first factor. This allows us to define the Dirac operator acting on vector spinors as follows

$$\begin{aligned} \not D:=h^{\alpha \beta }\partial _\alpha \cdot \nabla ^{SM\otimes \phi ^*TN}_{\partial _\beta }. \end{aligned}$$

We assume that the connection on $\phi ^*TP$ is metric and thus the operator $i\not D$ is also self-adjoint with respect to the $L^2$-norm. After these considerations we are ready to present the energy functional for Dirac-wave maps with curvature term

1.1 $$\begin{aligned} S\left( \phi ,{\tilde{\psi }},{\tilde{h}}\right) =\frac{1}{2}\int _{M}\left( |d\phi |^2+\left\langle {\tilde{\psi }},i{\tilde{\not D}}{\tilde{\psi }}\right\rangle -\frac{1}{6}\left\langle {\tilde{\psi }},R^P\left( {\tilde{\psi }},{\tilde{\psi }}\right) {\tilde{\psi }}\right\rangle \right) \text { }dV_{{\tilde{h}}}. \end{aligned}$$

In the last term the indices are contracted as follows

$$\begin{aligned} \left\langle {\tilde{\psi }},R^P\left( {\tilde{\psi }},{\tilde{\psi }}\right) {\tilde{\psi }}\right\rangle _{SM\otimes \phi ^*TP} =R_{IJKL}\left\langle {\tilde{\psi }}^I,{\tilde{\psi }}^K\right\rangle _{SM}\left\langle {\tilde{\psi }}^J,{\tilde{\psi }}^L\right\rangle _{SM}, \end{aligned}$$

which ensures that the energy functional is real-valued. We would like to point out that in the physics literature (see e.g. [1]) one usually considers Grassmann-valued spinors in the analysis of (1.1). However, we want to use methods from the geometric calculus of variation and due to this reason we are employing standard spinors.

The critical points of (1.1) can easily be calculated as

1.2 $$\begin{aligned} \tau (\phi )&=\frac{1}{2}h^{\alpha \beta }R^P\left( {\tilde{\psi }},i\partial _\alpha \cdot {\tilde{\psi }}\right) d\phi (\partial _\beta )-\frac{1}{12}\left\langle \left( \nabla R^P\right) ^\sharp \left( {\tilde{\psi }},{\tilde{\psi }}\right) {\tilde{\psi }},{\tilde{\psi }}\right\rangle , \end{aligned}$$

1.3 $$\begin{aligned} i{\tilde{\not D}}{\tilde{\psi }}&=\frac{1}{3}R^P\left( {\tilde{\psi }},{\tilde{\psi }}\right) {\tilde{\psi }}, \end{aligned}$$

where $\tau (\phi ):=-h^{\alpha \beta }\nabla _{\partial _\alpha }d\phi (\partial _\beta )$ represents the wave map operator. The solutions of (1.2), (1.3) are called *Dirac-wave maps with curvature term*.

We are able to provide the first existence result for the Dirac-wave map with curvature term system which is as follows:

### Theorem 1.1

Let $\tilde{g}_t$ be a smooth family of complete Riemannian metrics on $\Sigma ^{n-1}$, $N\in C^{\infty }(\mathbb {R}\times \Sigma )$ with $0< A\le N\le B<\infty$ and $(M^n,\tilde{h})=(\mathbb {R}\times \Sigma ,-N^2dt^2+\tilde{g}_t)$ be a globally hyperbolic Lorentzian spin manifold that satisfies the following condition: There exists a monotonically increasing smooth function $s:\mathbb {R}\rightarrow \mathbb {R}_+$ with $\int _0^\infty s^{-1} dt <\infty$, such that the conformal metric

$$\begin{aligned} h=(Ns)^{-2}\tilde{h}=-s^{-2}dt^2+g_t \end{aligned}$$

has bounded geometry, the metrics $g_t$ have a uniform Sobolev constant and

1.4 $$\begin{aligned} \Vert N\Vert _{C^k(g_t)}+\Vert \nabla _{\nu }N\Vert _{C^k(g_t)}+\Vert \mathrm {I\!I}\Vert _{C^k(g_t)}+s\Vert \mathrm {I\!I}\Vert _{L^\infty }\le C<\infty \end{aligned}$$

for all $k\in \mathbb {N}$. Here, $\nu$ is the future-directed unit normal of the hypersurfaces $\left\{ t\right\} \times \Sigma$ and $\mathrm {I\!I}$ is their second fundamental form.

Then if in addition, the Riemannian manifold (*P*, *G*) has bounded geometry, there exists for each $r\in \mathbb {N}$ with $r>\frac{n-1}{2}$ an $\varepsilon >0$ such that if the initial data $(\phi _0,\phi _1,\psi _0)$ for the system (1.2), (1.3) satisfies

1.5 $$\begin{aligned} \Vert \phi _0\Vert _{H^{r+1}(\tilde{g}_0)}+\Vert \phi _1\Vert _{H^r(\tilde{g}_0)}+\Vert \psi _0\Vert _{H^{r}(\tilde{g}_0)}<\varepsilon , \end{aligned}$$

the unique solution of the system (1.2), (1.3) with initial data $\phi |_{t=0}=\phi _0,\partial _t\phi |_{t=0}=\phi _1,\psi |_{t=0}=\psi _0$ exists for all times $t\in [0,\infty )$ and satisfies

$$\begin{aligned}&\phi \in C^0\left( [0,\infty ),H^{r+1}(\Sigma ,P)\right) \cap C^1\left( [0,\infty ),H^r(\Sigma ,P)\right) , \\&\psi \in C^0\left( [0,\infty ),H^{r}\left( M,SM\otimes \phi ^*TP\right) \right) \cap C^1\left( [0,\infty ),H^{r-1}\left( M,SM\otimes \phi ^*TP\right) \right) . \end{aligned}$$

### Remark 1.2

Under the same assumptions, the proof of Theorem 1.1 also implies global existence of *Dirac-wave maps*, which satisfy an equation slightly simpler than (1.2), (1.3). In this case, the second term on the right hand side of (1.2) and the right-hand side of (1.3) both vanish.

Along the line of Theorem 1.1 we obtain the following result for the wave map equation generalizing the results from [9, 10]:

### Theorem 1.3

Let $\tilde{g}_t$ be a smooth family of complete Riemannian metrics on $\Sigma ^{n-1}$, $N\in C^{\infty }(\mathbb {R}\times \Sigma )$ with $0< A\le N\le B<\infty$ and $(M^n,\tilde{h})=(\mathbb {R}\times \Sigma ,-N^2dt^2+\tilde{g}_t)$ be a globally hyperbolic Lorentzian manifold that satisfies the following condition: There exists a monotonically increasing smooth function $s:\mathbb {R}\rightarrow \mathbb {R}_+$ with $\int _0^\infty s^{-1} dt <\infty$, such that for the conformal metric

$$\begin{aligned} h=(Ns)^{-2}\tilde{h}=-s^{-2}dt^2+g_t \end{aligned}$$

the metrics $g_t$ admit a uniform Sobolev constant and

1.6 $$\begin{aligned} \Vert R^{g_t}\Vert _{C^k(g_t)}+\Vert N\Vert _{C^k(g_t)}+\Vert \nabla _{\nu }N\Vert _{C^k(g_t)}+\Vert \mathrm {I\!I}\Vert _{C^k(g_t)}\le C<\infty \end{aligned}$$

for all $k\in \mathbb {N}$. Here, $\nu$ is the future-directed unit normal of the hypersurfaces $\left\{ t\right\} \times \Sigma$ and $\mathrm {I\!I}$ is their second fundamental form.

Then if in addition, the Riemannian manifold (*P*, *G*) has bounded geometry, there exists for each $r\in \mathbb {N}$ with $r>\frac{n-1}{2}$ an $\varepsilon >0$ such that if the initial data $(\phi _0,\phi _1)$ for the wave map equation satisfies

1.7 $$\begin{aligned} \Vert \phi _0\Vert _{H^{r+1}(\tilde{g}_0)}+\Vert \phi _1\Vert _{H^r(\tilde{g}_0)}<\varepsilon , \end{aligned}$$

the unique wave map with initial data $\phi |_{t=0}=\phi _0,\partial _t\phi |_{t=0}=\phi _1$ exists for all times $t\in [0,\infty )$ and satisfies

$$\begin{aligned}&\phi \in C^0\left( [0,\infty ),H^{r+1}(\Sigma ,P)\right) \cap C^1\left( [0,\infty ),H^r(\Sigma ,P)\right) . \end{aligned}$$

### Remark 1.4

If we compare the assumptions of Theorems 1.1 and 1.3 we observe that one also has to control the $R_{0ij0}$-components of the curvature tensor in the result for Dirac-wave maps with curvature term. This curvature contribution appears when computing the curvature of the spinor bundle. This explains why we have to assume bounded geometry of the Lorentzian manifold in Theorem 1.1 but only bounded geometry of the Riemannian slices in Theorem 1.3.

In addition, we also have to demand a decay of the second fundamental form (1.4) in Theorem 1.1 which originates from the choice of a positive definite scalar product on the spinor bundle that is no longer metric.

### Remark 1.5

It seems that the class of Lorentzian manifolds that we are considering is the appropriate setting that guarantees a nice long-time behavior for solutions of various nonlinear wave equations with small initial data. We therefore think that our approach can be also used to establish existence results for a large class of other second order hyperbolic systems arising in mathematical physics.

### Example 1.6

There are many spacetimes that satisfy the assumptions in Theorems 1.1 and 1.3. The simplest class is the class of Robertson–Walker spacetimes $-dt^2+s^2(t)g$ with $s^{-1}\in L^1([0,\infty ))$ which already contains the de-Sitter space and the power-law inflation metric. More generally, it is believed that generic future geodesically complete solutions of the Einstein equation with positive cosmological constant $\Lambda >0$ satisfy the assumptions of our theorems.

Throughout this article we will employ the following notation: We will use small Greek letters $\alpha ~\beta ~\gamma$ for space-time indices, small Latin indices $i~j~k$ for spatial derivatives and capital Latin indices $I~J~K$ for indices on the Riemannian target. We will denote spatial derivative by $D$. Moreover, we will make use of the usual summation convention, that is we will always sum over repeated indices.

This article is organized as follows: In the second section we introduce a suitable conformal transformation that we will be using to prove our main results. In the third section we establish the necessary energy estimate which is the key tool to prove the main result.

## Conformal Euler–Lagrange equations

In the following we calculate how the energy functional (1.1) transforms under a certain conformal transformation of the metric on $M$.

### Lemma 2.1

If we transform the metric ${\tilde{h}}=(Ns)^2h$ and the vector spinors via

$$\begin{aligned} {\tilde{\psi }}:=(Ns)^{\frac{1-n}{2}}\psi \end{aligned}$$

the energy functional (1.1) acquires the form

2.1 $$\begin{aligned} S(\phi ,\psi ,h)=\frac{1}{2}\int _{M}\left( (Ns)^{n-2}|d\phi |^2+\left\langle \psi ,i\not D\psi \right\rangle -(Ns)^{2-n}\frac{1}{6}\left\langle \psi ,R^P(\psi ,\psi )\psi \right\rangle \right) \text { }dV_{h}. \end{aligned}$$

### Proof

Under a conformal change of the metric ${\bar{h}}=(Ns)^2h$ the volume elements transform as $\text { }dV_{{\bar{h}}}=(Ns)^n\text { }dV_h$. The Dirac operators transform as

$$\begin{aligned} {\bar{\not D}}\left( (Ns)^{-\frac{n-1}{2}}{\bar{\psi }}\right) =(Ns)^{-\frac{n+1}{2}}\overline{\not D\psi }. \end{aligned}$$

Note that the twisted Dirac operator $\not D$ transforms in the same way as the standard Dirac operator $\not \partial$ since the twist bundle $\phi ^*TP$ does not depend on the metric on $M$.

Inserting our choice for ${\tilde{\psi }}$ we get

$$\begin{aligned} \left\langle {\tilde{\psi }},i{\tilde{\not D}}{\tilde{\psi }}\right\rangle =(Ns)^{-n}\left\langle \psi ,i\not D\psi \right\rangle , \end{aligned}$$

which completes the proof. $\square$

For the sake of completeness we calculate the critical points of (2.1).

### Lemma 2.2

(Critical points) The critical points of (2.1) are given by

2.2 $$\begin{aligned} \Box _h\phi&=(n-2)(Ns)^{-1}\nabla _{\nabla (Ns)}\phi -\frac{1}{2}(Ns)^{2-n}h^{\alpha \beta }R^P\left( \psi ,i\partial _\alpha \cdot \psi \right) d\phi (\partial _\beta ) \nonumber \\&\quad +\frac{1}{12}(Ns)^{4-2n}\left\langle \left( \nabla R^P\right) ^\sharp (\psi ,\psi )\psi ,\psi \right\rangle , \nonumber \\ i\not D\psi&=(Ns)^{2-n}\frac{1}{3}R^P(\psi ,\psi )\psi . \end{aligned}$$

Here, $\Box :=-h^{\alpha \beta }\nabla _{\partial _\alpha }d\phi (\partial _\beta )$ denotes the wave map operator.

### Proof

First, we vary the vector spinors $\psi$ keeping the map $\phi$ fixed. More precisely, we consider a variation of $\psi :(-\varepsilon ,\varepsilon )\times M\rightarrow SM\otimes \phi ^*TP$ denoted by $\psi _\lambda$ satisfying $\frac{\nabla \psi _\lambda }{\partial \lambda }\big |_{\lambda =0}=\xi$. We calculate

$$\begin{aligned}&\frac{d}{d\lambda }\big |_{\lambda =0}\frac{1}{2}\int _M\left( \left\langle \psi _\lambda ,i\not D\psi _\lambda \right\rangle -(Ns)^{2-n}\frac{1}{6}\left\langle \psi _\lambda ,R^P\left( \psi _\lambda ,\psi _\lambda \right) \psi _\lambda \right\rangle \right) \text { }dV_{h}\\&\quad =\frac{1}{2}\int _M\left( \left\langle \xi ,i\not D\psi \right\rangle +\left\langle \psi ,i\not D\xi \right\rangle -(Ns)^{2-n}\frac{4}{6}\left\langle \xi ,R^P(\psi ,\psi )\psi \right\rangle \right) \text { }dV_{h}\\&\quad =\int _M\left( {\text {Re}}\left\langle \xi ,i\not D\psi \right\rangle -(Ns)^{2-n}\frac{1}{3}\left\langle \xi ,R^P(\psi ,\psi )\psi \right\rangle \right) \text { }dV_{h}, \end{aligned}$$

yielding the equation for the vector spinor.

Afterwards, we keep the vector spinors $\psi$ fixed and consider a variation of $\phi :(-\varepsilon ,\varepsilon )\times M\rightarrow P$ denoted by $\phi _\lambda$ satisfying $\frac{\partial \phi _\lambda }{\partial \lambda }\big |_{t=0}=\eta$. We calculate

$$\begin{aligned}&\frac{d}{d\lambda }\big |_{\lambda =0}\frac{1}{2}\int _M(Ns)^{n-2}|d\phi _\lambda |^2\text { }dV_h\\&\quad =\int _M(Ns)^{n-2}\left\langle \nabla \frac{\partial \phi _\lambda }{\partial \lambda },d\phi _\lambda \right\rangle \text { }dV_h\big |_{\lambda =0} \\&\quad =\int _M\left( (Ns)^{n-2}\left\langle \eta ,\Box \phi \right\rangle -(n-2)(Ns)^{n-3}\nabla (Ns)\left\langle \eta ,\nabla \phi \right\rangle \right) \text { }dV_h. \end{aligned}$$

Moreover, we have

$$\begin{aligned} \frac{d}{d\lambda }&\big |_{\lambda =0}\frac{1}{2}\int _M\left( \left\langle \psi ,i\not D\psi \right\rangle -(Ns)^{2-n}\frac{1}{6}\left\langle \psi ,R^P(\psi ,\psi )\psi \right\rangle \right) \text { }dV_{h} \\&=\frac{1}{2}\int _Mh^{\alpha \beta }\left\langle \psi ,i R^{P}\left( \eta ,d\phi (\partial _\alpha )\right) \partial _\beta \cdot \psi \right\rangle \text { }dV_h\\&\quad +\frac{1}{12}\int _M(Ns)^{2-n}\left\langle \left\langle \left( \nabla R^P\right) ^\sharp (\psi ,\psi )\psi ,\psi \right\rangle ,\eta \right\rangle \text { }dV_h, \end{aligned}$$

where we used the equation for $\psi$. Finally, we have

$$\begin{aligned} h^{\alpha \beta }\left\langle \psi ,i R^{P}\left( \eta ,d\phi (\partial _\alpha )\right) \partial _\beta \cdot \psi \right\rangle =h^{\alpha \beta }\left\langle \eta , R^{P}(\psi ,i\partial _\alpha \cdot \psi )d\phi (\partial _\beta )\right\rangle , \end{aligned}$$

which completes the proof. $\square$

We want to turn the system (2.2) into a system of two wave-type equations. To this end we recall the following Weitzenböck formula for the twisted Dirac operator $\not D$.

### Lemma 2.3

The square of the twisted Dirac operator $\not D$ satisfies

2.3 $$\begin{aligned} \not D^2=\Box +\frac{\mathrm {scal}^M}{4}+\frac{1}{2}h^{\alpha \gamma }h^{\beta \delta }\partial _\alpha \cdot \partial _\beta \cdot R^P\left( d\phi \left( \partial _\gamma \right) ,d\phi \left( \partial _\delta \right) \right) \psi . \end{aligned}$$

### Proof

For a proof the we refer to [19, Theorem 8.17]. $\square$

Making use of (2.3) we obtain the following rescaled Euler–Lagrange equations

2.4 $$\begin{aligned} \Box _h\phi&=(n-2)(Ns)^{-1}\nabla _{\nabla (Ns)}\phi -\frac{1}{2}(Ns)^{2-n}h^{\alpha \beta }R^P(\psi ,i\partial _\alpha \cdot \psi )d\phi (\partial _\beta ) \nonumber \\&\quad +\frac{1}{12}(Ns)^{4-2n}\left\langle \left( \nabla R^P\right) ^\sharp (\psi ,\psi )\psi ,\psi \right\rangle , \end{aligned}$$

2.5 $$\begin{aligned} \Box _h\psi&=-\frac{\mathrm {scal}^M}{4}\psi -\frac{1}{2}h^{\alpha \gamma }h^{\beta \delta }\partial _\alpha \cdot \partial _\beta \cdot R^P\left( d\phi \left( \partial _\gamma \right) ,d\phi \left( \partial _\delta \right) \right) \psi \nonumber \\&\quad +\frac{i}{3}\nabla \left( R^P(\psi ,\psi )(Ns)^{2-n}\right) \cdot \psi \nonumber \\&\quad +\frac{1}{9}(Ns)^{4-2n}R^P(\psi ,\psi )R^P(\psi ,\psi )\psi , \end{aligned}$$

where $\Box _h$ denotes the corresponding wave-operator.

In terms of local coordinates this system acquires the form ($I=1,\ldots ,\dim P$)

$$\begin{aligned}&s^2\partial ^2_t\phi ^I+s\dot{s}\partial _t\phi ^I+\frac{1}{2}s^2{\text {tr}}\dot{g}\partial _t\phi ^I+D^*D\phi ^I \\&\quad =-(n-2)\dot{s}s\partial _t\phi ^I-(n-2)s^2N^{-1}\partial _tN\partial _t\phi ^I +(n-2)N^{-1}D_{\partial _i}ND_{\partial _i}\phi ^I \\&\qquad -\frac{1}{2}(Ns)^{2-n}R^I_{JKL}h^{\alpha \beta }\left\langle \psi ^K,i\partial _\alpha \cdot \psi ^L\right\rangle \partial _\beta \phi ^J\\&\qquad +\frac{1}{12}(Ns)^{4-2n}G^{IJ}\nabla _JR_{KLMN}\left\langle \psi ^K,\psi ^M\right\rangle \left\langle \psi ^L,\psi ^N\right\rangle ,\\&s^2\nabla ^2_t\psi ^I+s\dot{s}\nabla _t\psi ^I+\frac{1}{2}s^2{\text {tr}}\dot{g}\nabla _t\psi ^I+D^*D\psi ^I \\&\quad =-\frac{\mathrm {scal}^M}{4}\psi ^I -\frac{1}{2}h^{\alpha \gamma }h^{\beta \delta }R^I_{JKL}\partial _\gamma \phi ^K\partial _\delta \phi ^L\partial _\alpha \cdot \partial _\beta \cdot \psi ^J\\&\qquad +\frac{i}{3}h^{\alpha \beta }\nabla _{\partial _\alpha }\left( (Ns)^{2-n}R^I_{JKL}\left\langle \psi ^J,\psi ^L\right\rangle \partial _{y^I}\right) \partial _\beta \cdot \psi ^K\\&\qquad +\frac{1}{9}(Ns)^{4-2n}R^I_{JKL}R^K_{MRS}\left\langle \psi ^J,\psi ^L\right\rangle \left\langle \psi ^M,\psi ^S\right\rangle \psi ^R. \end{aligned}$$

### Remark 2.4

Note that the system (1.2), (1.3) on the manifold $(M,{\tilde{h}})$ is equivalent to the system (2.2) on the conformally transformed manifold $(M,h)$. From now on we will use the system (2.4), (2.5) which follows from (2.2).

## The energy estimate

In this section we first develop several formulas that are useful in the study of energy estimates for sections in arbitrary vector bundles. Later on, we will apply these techniques to the cases of wave maps and Dirac-wave maps with curvature term.

### Energies on general vector bundles

Let $\Sigma ^n$ be a manifold, $s:\mathbb {R}\rightarrow \mathbb {R}_+$ smooth and $g_t$, $t\in \mathbb {R}$ be a smooth family of Riemannian metrics on $\Sigma$. We consider the (globally hyperbolic) Lorentzian manifold

$$\begin{aligned} (M,h)=\left( \mathbb {R}\times \Sigma , -s(t)^{-2}dt^2+g_t\right) . \end{aligned}$$

The non-vanishing Christoffel symbols of this metric are given by

$$\begin{aligned} \Gamma (h)_{00}^0=-\frac{{\dot{s}}}{s},\qquad \Gamma (h)_{ij}^0=\frac{1}{2}s^2{\dot{g}}_{ij},\qquad \Gamma (h)_{i0}^j=\Gamma (h)_{0i}^j=\frac{1}{2}g^{jk}{\dot{g}}_{ik},\qquad \Gamma (h)_{ij}^k=\Gamma (g)_{ij}^k. \end{aligned}$$

Let *V* be a Riemannian vector bundle over *M* which is equipped with a metric connection $\nabla$. As usual, iterating yields a map

$$\begin{aligned} \nabla ^k:\Gamma (V)\rightarrow \Gamma \left( T^*M^{\otimes k}\otimes V\right) . \end{aligned}$$

We define the associated wave operator $\Box :\Gamma (V)\rightarrow \Gamma (V)$ by the sign convention such that

$$\begin{aligned} \Box \xi =-h^{\alpha \beta }\nabla ^2_{\alpha \beta }\xi \end{aligned}$$

for $\xi \in \Gamma (V)$. The covariant derivative $\nabla$ restricts for each $t\in \mathbb {R}$ to the spatial covariant derivative *D* which yields a map

$$\begin{aligned} D:\Gamma (V)\rightarrow \Gamma \left( \pi ^*\left( T^*\Sigma \right) \otimes V\right) , \end{aligned}$$

where $\pi :M\rightarrow \Sigma$ is the canonical projection. In the following, we will write $T^*\Sigma$ instead of $\pi ^*(T^*\Sigma )$ for notational convenience. The covariant derivative naturally extends as a map

$$\begin{aligned} D:\Gamma \left( T^*\Sigma ^{\otimes k}\otimes V\right) \rightarrow \Gamma \left( T^*\Sigma ^{\otimes k+1}\otimes V\right) \end{aligned}$$

by defining

$$\begin{aligned} \left( D_X\xi \right) \left( X_1,\ldots ,X_k\right) ={}^{V}\nabla _X\left( \xi \left( X_1,\ldots ,X_k\right) \right) -\sum _{i=1}^k\xi \left( X_1,\ldots ,D_XX_i,\ldots ,X_k\right) , \end{aligned}$$

where for each $t\in \mathbb {R}$, $D_XX_i$ denotes the covariant derivative of $g_t$. Furthermore, we define its formal adjoint $D^*:\Gamma (T^*\Sigma ^{\otimes k+1}\otimes V)\rightarrow \Gamma (T^*\Sigma ^{\otimes k}\otimes V)$ by

$$\begin{aligned} D^*\xi \left( X_1,\ldots ,X_k\right) =-g^{ij}D_{\partial _i}\xi \left( \partial _j,X_1,\ldots ,X_k\right) . \end{aligned}$$

Finally, we define a covariant time-derivative $\nabla _t:\Gamma (T^*\Sigma ^{\otimes k}\otimes V)\rightarrow \Gamma (T^*\Sigma ^{\otimes k}\otimes V)$ by

$$\begin{aligned} \left( \nabla _t\xi \right) \left( X_1,\ldots ,X_k\right) =\nabla ^V_{\partial _t}\left( \xi \left( X_1,\ldots ,X_k\right) \right) -\sum _{i=1}^k\xi \left( X_1,\ldots ,\nabla _{\partial _t}X_i,\ldots ,X_k\right) . \end{aligned}$$

Observe that this definition makes sense as $\nabla _{\partial _t}X_i\in \Gamma (TM)$ is always tangential to $\Sigma$ due to the structure of the metric *h*. A quick computation shows that the wave operator can be written as

3.1 $$\begin{aligned} \Box \xi =s^2\nabla _t\nabla _t\xi +{\dot{s}}s\nabla _t\xi +\frac{1}{2}s^2{\text {tr}}_g{\dot{g}}\cdot \nabla _t\xi +D^*D\xi . \end{aligned}$$

#### Lemma 3.1

We use the notations from above. Let $\langle .,.\rangle$ be the natural *t*-dependent scalar product induced on $\Gamma (T^*\Sigma ^{\otimes k}\otimes V)$. Let $\xi ,\eta \in \Gamma (V)$. Then we have the product rule

$$\begin{aligned} \partial _t\left\langle D^k\xi ,D^k\eta \right\rangle = \left\langle \nabla _t D^k\xi ,D^k\eta \right\rangle +\left\langle D^k\xi ,\nabla _t D^k\eta \right\rangle . \end{aligned}$$

#### Proof

Let $t_0$ be a fixed time and let $\left\{ e_1,\ldots ,e_n\right\}$ be an orthonormal basis of $(\Sigma ,g_{t_0})$ at $x\in \Sigma$. Then the scalar product can also be written as

$$\begin{aligned} \left\langle D^k\xi ,D^k\eta \right\rangle (t_0,x)=\sum _{i_1,\ldots ,i_k=1}^n \left\langle D^k_{e_{i_1},\ldots ,e_{i_k}}\xi ,D^k_{e_{i_1},\ldots ,e_{i_k}}\eta \right\rangle (t_0,x), \end{aligned}$$

where the scalar products on the right hand side are the ones on *V*. Now think of $\left\{ e_1,\ldots ,e_n\right\}$ as an orthonormal system in $T_{(t_0,x)}M$. Parallel transport along the curve $t\mapsto (t,x)$ yields orthonormal systems $\left\{ e_1,\ldots ,e_n\right\}$ for each $T_{(t,x)}M$. Because $\nabla _{\partial _t}\partial _t=-\frac{{\dot{s}}}{s}\partial _t$, $h(\partial _t,e_i)\equiv 0$ for each $i\in \left\{ 1,\ldots ,n\right\}$ so that we in fact obtain orthonormal bases of $T_x\Sigma$ with respect to the metric $g_t$ for each $t\in \mathbb {R}$. Therefore we get

$$\begin{aligned} \partial _t\left\langle D^k\xi ,D^k\eta \right\rangle&=\sum _{i_1,\ldots ,i_k=1}^n \left\langle \nabla _t \left( D^k_{e_{i_1},\ldots ,e_{i_k}}\xi \right) ,D^k_{e_{i_1},\ldots ,e_{i_k}}\eta \right\rangle \\&\quad +\sum _{i_1,\ldots ,i_k=1}^n\left\langle D^k_{e_{i_1},\ldots ,e_{i_k}}\xi ,\nabla _t \left( D^k_{e_{i_1},\ldots ,e_{i_k}}\eta \right) \right\rangle \\&=\sum _{i_1,\ldots ,i_k=1}^n \left\langle \nabla _t D^k_{e_{i_1},\ldots ,e_{i_k}}\xi ,D^k_{e_{i_1},\ldots ,e_{i_k}}\eta \right\rangle +\sum _{i_1,\ldots ,i_k=1}^n\left\langle D^k_{e_{i_1},\ldots ,e_{i_k}}\xi ,\nabla _t D^k_{e_{i_1},\ldots ,e_{i_k}}\eta \right\rangle \\&=\left\langle \nabla _t D^k\xi ,D^k\eta \right\rangle +\left\langle D^k\xi ,\nabla _t D^k\eta \right\rangle . \end{aligned}$$

$$\square $$

#### Remark 3.2

In the above lemma, the special structure of the metric *h* is essential.

For a section $\xi \in \Gamma (V)$, we now define the *k*th energy density

$$\begin{aligned} e_k(\xi )=s^2\left| D^k\nabla _t\xi \right| ^2+\left| D^{k+1}\xi \right| ^2, \end{aligned}$$

which is a nonnegative function on *M* and the *k*th energy as

$$\begin{aligned} E_k(\xi )(t)=\int _{\Sigma }e_k(\xi )\text { }dV_{g_t}, \end{aligned}$$

where $\text { }dV_{g_t}$ is the volume element of the metric $g_t$ and the integral has to be understood as an integral over $\left\{ t\right\} \times \Sigma$.

#### Proposition 3.3

Let $\xi \in \Gamma (V)$ be spacelike compactly supported. Then its *k*th energy satisfies the evolution equation

$$\begin{aligned} \frac{d}{dt} E_k(\xi )&=2\int _{\Sigma }\left\langle D^k\Box \xi ,D^k\nabla _t\xi \right\rangle \text { }dV_{g_t}+\frac{1}{2}\int _{\Sigma }\left( \left| D^{k+1}\xi \right| ^2-s^2\left| D^k\nabla _t\xi \right| ^2\right) {\text {tr}}_g{\dot{g}}\text { }dV_{g_t}\\&\qquad +2s^2\int _{\Sigma }\left\langle D^k\nabla _t\xi ,\left[ \nabla _t,D^k\right] \nabla _t\xi \right\rangle \text { }dV_{g_t}+2\int _{\Sigma }\left\langle D^k\nabla _t\xi ,\left[ D^*D,D^k\right] \xi \right\rangle \text { }dV_{g_t}\\&\qquad +2\int _{\Sigma }\left\langle D^{k+1}\xi ,\left[ \nabla _t,D^{k+1}\right] \xi \right\rangle \text { }dV_{g_t}. \end{aligned}$$

#### Proof

We compute

$$\begin{aligned} \frac{d}{dt}E_k(\xi )&=2{\dot{s}}s\int _{\Sigma }\left| D^k\nabla _t\xi \right| ^2\text { }dV_{g_t}+\frac{1}{2}\int _{\Sigma }e_k(\xi ){\text {tr}}_g{\dot{g}}\text { }dV_{g_t}\\&\quad +2s^2\int _{\Sigma }\left\langle \nabla _tD^k\nabla _t\xi ,D^k\nabla _t\xi \right\rangle \text { }dV_{g_t}+2\int _{\Sigma }\left\langle \nabla _tD^{k+1}\xi ,D^{k+1}\xi \right\rangle \text { }dV_{g_t}\\&=2{\dot{s}}s\int _{\Sigma }\left| D^k\nabla _t\xi \right| ^2\text { }dV_{g_t}+\frac{1}{2}\int _{\Sigma }e_k(\xi ){\text {tr}}_g{\dot{g}}\text { }dV_{g_t}\\&\quad +2s^2\int _{\Sigma }\left\langle D^k\nabla _t\nabla _t\xi ,D^k\nabla _t\xi \right\rangle \text { }dV_{g_t}+2s^2\int _{\Sigma }\left\langle \left[ \nabla _t,D^k\right] \nabla _t\xi ,D^k\nabla _t\xi \right\rangle \text { }dV_{g_t}\\&\quad +2\int _{\Sigma }\left\langle D^{k+1}\nabla _t\xi ,D^{k+1}\xi \right\rangle \text { }dV_{g_t}+2\int _{\Sigma }\left\langle \left[ \nabla _t,D^{k+1}\right] \xi ,D^{k+1}\xi \right\rangle \text { }dV_{g_t}. \end{aligned}$$

Moreover, we have

$$\begin{aligned} 2\int _{\Sigma }\left\langle D^{k+1}\nabla _t\xi ,D^{k+1}\xi \right\rangle \text { }dV_{g_t}&=2\int _{\Sigma }\left\langle D^k\nabla _t\xi ,D^*D^{k+1}\xi \right\rangle \text { }dV_{g_t}\\&= 2\int _{\Sigma }\left\langle D^k\nabla _t\xi ,D^k D^*D\xi \right\rangle \text { }dV_{g_t}\\&\quad +2\int _{\Sigma }\left\langle D^k\nabla _t\xi ,\left[ D^{*}D,D^k\right] \xi \right\rangle \text { }dV_{g_t}. \end{aligned}$$

The statement follows by combining both equalities and using (3.1). $\square$

In order to derive energy estimates for the vector spinors we have to take into account the special structure of the scalar product of the spinor bundle $SM$. The natural geometric scalar product which is invariant under the spin group is not positive definite and thus not a good candidate for analytic purposes. To obtain a positive definite scalar product one has to Clifford multiply the second factor with the timelike unit vector field $e_0$, see [3], which in our setup is given by $e_0=s\partial _t$. Using our geometric setup we find

$$\begin{aligned} \nabla _te_0=0,\qquad \nabla _ie_0=\frac{1}{2}sg^{jk}\dot{g}_{ik}\partial _{x^j}. \end{aligned}$$

Hence, we define the following $k$th energy density for the vector spinors $\psi$

$$\begin{aligned} e_k(\psi )=s^2\left\langle D^k\nabla _t\psi ,e_0\cdot D^k\nabla _t\psi \right\rangle +\left\langle D^{k+1}\psi ,e_0\cdot D^{k+1}\psi \right\rangle , \end{aligned}$$

which is a nonnegative function. In the following we will always employ the positive definite scalar product without mentioning it explicitly. However, since $e_0$ is not parallel with respect to the spatial coordinates the $k$th energy of the spinor satisfies

3.2 $$\begin{aligned} \frac{d}{dt} E_k(\psi )&=2\int _{\Sigma }\left\langle D^k\Box \psi , D^k\nabla _t\psi \right\rangle \text { }dV_{g_t}+\frac{1}{2}\int _{\Sigma }\left( \left| D^{k+1}\psi \right| ^2-s^2\left| D^k\nabla _t\psi \right| ^2\right) {\text {tr}}_g{\dot{g}}\text { }dV_{g_t}\nonumber \\&\quad +2s^2\int _{\Sigma }\left\langle D^k\nabla _t\psi ,\left[ \nabla _t,D^k\right] \nabla _t\psi \right\rangle \text { }dV_{g_t}+2\int _{\Sigma }\left\langle D^k\nabla _t\psi ,\left[ D^*D,D^k\right] \psi \right\rangle \text { }dV_{g_t}\nonumber \\&\quad +2\int _{\Sigma }\left\langle D^{k+1}\psi ,\left[ \nabla _t,D^{k+1}\right] \psi \right\rangle \text { }dV_{g_t} +2\int _{\Sigma }\left\langle D^{k}\psi ,e_0\cdot \left( D^*e_0\right) \cdot D^{k+1}\psi \right\rangle \text { }dV_{g_t}. \end{aligned}$$

#### Lemma 3.4

Let $\xi \in \Gamma (T^*\Sigma ^{\otimes k}\otimes V)$. Then we have the identity

$$\begin{aligned} \left( \left[ \nabla _t,D\right] \xi \right) \left( X,X_1,\ldots ,X_k\right)&=R^V_{\partial _t,X}(\xi (X_1,\ldots ,X_k))-\frac{1}{2}D_{{\dot{g}}(X)}\xi (X_1,\ldots ,X_k)\\&\qquad +\frac{1}{2}\sum _{i=1}^k\xi \left( X_1,\ldots ,D_X{\dot{g}}(X_i),\ldots ,X_k\right) . \end{aligned}$$

#### Proof

At first, we compute

$$\begin{aligned}&\left( \nabla _tD\xi \right) (X,X_1,\ldots ,X_k)\\&\quad =\nabla ^V_{\partial _t}(D\xi (X,X_1,\ldots ,X_k))-(D\xi )(\nabla _{\partial _t}X,X_1,\ldots ,X_k)\\&\qquad -\sum _{i=1}^k(D\xi )(X,X_1,\ldots ,\nabla _{\partial _t}X_i,\ldots ,X_k)\\&\quad =\nabla ^V_{\partial _t}\Big (\nabla ^V_X(\xi (X_1,\ldots ,X_k))- \sum _{i=1}^k\xi (X_1,\ldots ,D_XX_i,\ldots X_k)\Big )\\&\quad \quad -\left( D_{\nabla _{\partial _t}X}\xi \right) (X_1,\ldots ,X_k) -\sum _{i=1}^k\left( D_X\xi \right) (X_1,\ldots ,\nabla _{\partial _t}X_i,\ldots ,X_k)\\&\quad =\nabla ^V_{\partial _t}\left( \nabla ^V_X(\xi (X_1,\ldots ,X_k))\right) -\sum _{i=1}^k\left( \nabla _t\xi \right) (X_1,\ldots ,D_XX_i,\ldots X_k)\\&\quad \quad -\sum _{i=1}^k\xi \left( X_1,\ldots ,\nabla _{\partial _t}D_XX_i,\ldots X_k\right) -\sum _{\begin{array}{c} i,j=1\\ i\ne j \end{array}}^n \xi \left( X_1,\ldots ,D_XX_i,\ldots ,\nabla _{\partial _t}X_j,\ldots X_k\right) \\&\quad \quad -\left( D_{\nabla _{\partial _t}X}\xi \right) (X_1,\ldots ,X_k) -\sum _{i=1}^k\left( D_X\xi \right) \left( X_1,\ldots ,\nabla _{\partial _t}X_i,\ldots ,X_k\right) . \end{aligned}$$

Similarly, we find

$$\begin{aligned}&\left( D\left( \nabla _t\xi \right) \right) (X,X_1,\ldots ,X_k)\\&\quad =\nabla ^V_X\left( \left( \nabla _t\xi \right) \left( X_1,\ldots ,X_k\right) \right) -\sum _{i=1}^k\left( \nabla _t\xi \right) (X_1,\ldots ,D_XX_i,\ldots ,X_k)\\&\quad =\nabla ^V_X\left( \nabla ^V_{\partial _t}\left( \xi \left( X_1,\ldots ,X_k\right) \right) -\sum _{i=1}^k\xi \left( X_1,\ldots ,\nabla _{\partial _t}X_i,\ldots ,X_k\right) \right) \\&\quad \quad -\sum _{i=1}^k(\nabla _t\xi )(X_1,\ldots ,D_XX_i,\ldots ,X_k)\\&\quad =\nabla ^V_X\left( \nabla ^V_{\partial _t}(\xi (X_1,\ldots ,X_k))\right) -\sum _{i=1}^k\left( D_X\xi \right) \left( X_1,\ldots ,\nabla _{\partial _t}X_i,\ldots X_k\right) \\&\quad \quad -\sum _{i=1}^k\xi \left( X_1,\ldots ,D_X\nabla _{\partial _t}X_i,\ldots X_k\right) -\sum _{\begin{array}{c} i,j=1\\ i\ne j \end{array}}^n \xi \left( X_1,\ldots ,D_XX_i,\ldots ,\nabla _{\partial _t}X_j,\ldots X_k\right) \\&\quad \quad -\sum _{i=1}^k\left( \nabla _t\xi \right) \left( X_1,\ldots ,D_XX_i,\ldots ,X_k\right) . \end{aligned}$$

Summing up, we obtain

$$\begin{aligned} \left( \left[ \nabla _t,D\right] \xi \right) (X,X_1,\ldots ,X_k)&=\nabla ^V_{\partial _t}\left( \nabla ^V_X(\xi (X_1,\ldots ,X_k))\right) -\nabla ^V_X\left( \nabla ^V_{\partial _t}(\xi (X_1,\ldots ,X_k))\right) \\&\quad -\sum _{i=1}^k\xi \left( X_1,\ldots ,\nabla _{\partial _t}D_XX_i-D_X\nabla _{\partial _t}X_i,\ldots X_k\right) \\&\quad -\left( D_{\nabla _{\partial _t}X}\xi \right) (X_1,\ldots ,X_k). \end{aligned}$$

As this expression is tensorial in *X* and the $X_i$, we may assume that the components of these vector fields with respect to a chart of $\Sigma$ are independent of time. By raising an index with respect to $g_t$ we can think of ${\dot{g}}_t$ as an endomorphism on $T^*\Sigma$. Then the formulas for the Christoffel symbols imply $\nabla _{\partial _t}X=\nabla _X\partial _t=\frac{1}{2}{\dot{g}}(X)$ and the same holds for $X_i$. Moreover, we have $[\partial _t,X]=[\partial _t,X_i]=0$ and

$$\begin{aligned} \nabla _{\partial _t}D_XX_i-D_X\nabla _{\partial _t}X_i=\frac{1}{2}{\dot{g}}\left( D_XX_i\right) -D_X\left( {\dot{g}}\left( X_i\right) \right) =-\frac{1}{2}D_X{\dot{g}}\left( X_i\right) . \end{aligned}$$

By putting these facts together, we immediately get the statement of the Lemma. $\square$

In the following we will often make use of the so-called $\star$ notation. More precisely, we will use a $\star$ to denote various contractions between the objects involved.

We apply the general formula from above in the case where we have a map $\phi \in C^{\infty }(M,P)$, $[\nabla _t,D]\phi \in \Gamma (T^*\Sigma \otimes \phi ^*TP)$ and we get

$$\begin{aligned} \left( \left[ \nabla _t,D\right] \phi \right) (X)=-\frac{1}{2}d\phi \left( {\dot{g}}(X)\right) . \end{aligned}$$

More generally, if $E=\phi ^*TP$ in the Lemma above, we get

$$\begin{aligned} \left( \left[ \nabla _t,D\right] \xi \right) (X,X_1,\ldots ,X_k)&=R^P(d\phi (\partial _t),d\phi (X))(\xi (X_1,\ldots ,X_k))-\frac{1}{2}D_{{\dot{g}}(X)}\xi (X_1,\ldots ,X_k)\\&\quad +\frac{1}{2}\sum _{i=1}^k\xi \left( X_1,\ldots ,D_X{\dot{g}}(X_i),\ldots ,X_k\right) \\&=R^P\left( \nabla _t\phi ,D_X\phi \right) \left( \xi (X_1,\ldots ,X_k)\right) -\frac{1}{2}D_{{\dot{g}}(X)}\xi (X_1,\ldots ,X_k)\\&\quad +\frac{1}{2}\sum _{i=1}^k\xi \left( X_1,\ldots ,D_X{\dot{g}}(X_i),\ldots ,X_k\right) . \end{aligned}$$

By an iteration argument, we find

$$\begin{aligned} \left[ \nabla _t,D^k\right] \nabla _t\phi&=\sum _{\sum l_i+\sum {m_j}=k-1}{}^{G}\nabla ^{l_1}R^P\star \underbrace{D^{m_1+1}\phi \star \cdots \star D^{m_{l_1}+1}\phi }_{l_1-\text {times}}\star D^{l_2}\nabla _t\phi \star D^{l_3+1}\phi \star D^{l_4}\nabla _t\phi \\&\quad +\sum _{l=0}^{k-1}D^{k-l}{\dot{g}}\star D^{l}\nabla _t\phi ,\\ \left[ \nabla _t,D^{k+1}\right] \phi&=\sum _{\sum l_i+\sum {m_j}=k-1}{}^{G}\nabla ^{l_1}R^N\star \underbrace{D^{m_1+1}\phi \star \cdots \star D^{m_{l_1}+1}\phi }_{l_1-\text {times}}\star D^{l_2}\nabla _t\phi \star D^{l_3+1}\phi \star D^{l_4+1}\phi \\&\quad +\sum _{l=0}^{k}D^{k-l}{\dot{g}}\star D^{l+1}\phi . \end{aligned}$$

In the case of a vector spinor $\psi \in \Gamma (SM\otimes \phi ^*TP)$, the formulas are

$$\begin{aligned} \left( \left[ \nabla _t,D\right] \psi \right) (X)=R^{SM}({\partial _t,X})\cdot \psi +R^P\left( \nabla _t\phi ,D_X\phi \right) \psi +\frac{1}{2}D\phi \left( {\dot{g}}(X)\right) \end{aligned}$$

and

$$\begin{aligned}&\left( \left[ \nabla _t,D\right] \xi \right) (X,X_1,\ldots ,X_k)\\&\quad =R^{SM}(\partial _t,X)\cdot \xi +R^P\left( \nabla _t\phi ,D_X\phi \right) (\xi (X_1,\ldots ,X_k))\\&\quad \quad -\frac{1}{2}D_{{\dot{g}}(X)}\xi (X_1,\ldots ,X_k)+\frac{1}{2}\sum _{i=1}^k\xi (X_1,\ldots ,D_X{\dot{g}}(X_i),\ldots ,X_k). \end{aligned}$$

By iterating the formula from above, we get

$$\begin{aligned} \left[ \nabla _t,D^k\right] \nabla _t\psi&= \sum _{l=0}^{k-1}D^l\left( R^{SM}(\partial _t,.)\right) \star D^{k-1-l}\nabla _t\psi +\sum _{l=0}^{k-1}D^{k-l}{\dot{g}}\star D^{l+1}\nabla _t\psi \\&\quad +\sum _{\sum l_i+\sum {m_j}=k-1}{}^{G}\nabla ^{l_1}R^P\star \underbrace{D^{m_1+1}\phi \star \cdots \star D^{m_{l_1}+1}\phi }_{l_1-\text {times}}\star D^{l_2}\nabla _t\phi \star D^{l_3+1}\phi \star D^{l_4}\nabla _t\psi \end{aligned}$$

and

$$\begin{aligned} \left[ \nabla _t,D^{k+1}\right] \psi&=\sum _{l=0}^{k}D^l\left( R^{SM}(\partial _t,.)\right) \star D^{k-l}\psi +\sum _{l=0}^{k}D^{k-l+1}{\dot{g}}\star D^{l+1}\psi \\&\quad +\sum _{\sum l_i+\sum {m_j}=k}{}^{G}\nabla ^{l_1}R^P\star \underbrace{D^{m_1+1}\phi \star \cdots \star D^{m_{l_1}+1}\phi }_{l_1-\text {times}}\star D^{l_2}\nabla _t\phi \star D^{l_3+1}\phi \star D^{l_4}\psi . \end{aligned}$$

#### Lemma 3.5

Let $\xi \in \Gamma (T^*\Sigma ^{\otimes k}\otimes V)$. Then we have the identity

$$\begin{aligned} \left( \left[ D^*D,D\right] \xi \right) (X,X_1,\ldots ,X_k)&=-D_{\mathrm {Ric}(X)}\xi (X_1,\ldots ,X_k)\\&\quad -2\sum _{j=1}^kD_{e_i}\xi (X_1,\ldots R_{X,e_i}X_j,\ldots ,X_k)\\&\quad + D_{e_i}R^V_{X,e_i}(\xi (X_1,\ldots ,X_k)) +2R^V_{X,e_i}\left( D_{e_i}\xi (X_1,\ldots ,X_k)\right) \\&\quad -\sum _{j=1}^k\xi \left( X_1,\ldots D_{e_i}R_{X,e_i}X_j,\ldots ,X_k\right) , \end{aligned}$$

where $\left\{ e_i\right\} _{1\le i\le n-1}$ is a local orthonormal frame. Here and throughout the proof, we sum over *i*.

#### Proof

A direct computation yields

$$\begin{aligned}&\left( \left[ D^*D,D\right] \xi \right) (X,X_1,\ldots ,X_k)\\&\quad =D^3_{X,e_i,e_i}\xi (X_1,\ldots ,X_k)-D^3_{e_i,e_i,X}\xi (X_1,\ldots ,X_k)\\&\quad =D^3_{X,e_i,e_i}\xi (X_1,\ldots ,X_k)-D^3_{e_i,X,e_i}\xi (X_1,\ldots ,X_k)\\&\quad \quad +D^3_{e_i,X,e_i}\xi (X_1,\ldots ,X_k)-D^3_{e_i,e_i,X}\xi (X_1,\ldots ,X_k)\\&\quad =R_{X,e_i}D\xi (e_i,X_1,\ldots ,X_k)+D_{e_i}R_{X,e_i}\xi (X_1,\ldots ,X_k)\\&\quad =-D_{\mathrm {Ric}(X)}\xi (X_1,\ldots ,X_k) -\sum _{j=1}^kD_{e_i}\xi \left( X_1,\ldots R_{X,e_i}X_j,\ldots ,X_k\right) \\&\quad \quad +R_{X,e_i}(D_{e_i}\xi (X_1,\ldots ,X_k))+D_{e_i} \left( R^V\circ \xi \right) \left( X,e_i,X_1,\ldots ,X_k\right) \\&\quad \quad -D_{e_i}\sum _{j=1}^k\xi \left( X_1,\ldots R_{X,e_i}X_j,\ldots ,X_k\right) \\&\quad =-D_{\mathrm {Ric}(X)}\xi (X_1,\ldots ,X_k)-2\sum _{j=1}^kD_{e_i}\xi \left( X_1,\ldots R_{X,e_i}X_j,\ldots ,X_k\right) \\&\quad \quad + D_{e_i}R^V_{X,e_i}(\xi (X_1,\ldots ,X_k)) +2R_{X,e_i}\left( D_{e_i}\xi (X_1,\ldots ,X_k)\right) \\&\quad \quad -\sum _{j=1}^k\xi \left( X_1,\ldots D_{e_i}R_{X,e_i}X_j,\ldots ,X_k\right) . \end{aligned}$$

Here, $R^V\circ \xi \in \Gamma (T^*\Sigma ^{\otimes k+2}\otimes V)$ is defined as

$$\begin{aligned} \left( R^V\circ \xi \right) (X,Y,X_1,\ldots ,X_k)=R_{X,Y}^V(\xi (X_1,\ldots ,X_k)) \end{aligned}$$

and $DR^{V}$ is the covariant derivative of the curvature endomorphism on *V* restricted to vectors tangential to $\Sigma$. In other words,

$$\begin{aligned} D_{X}R^V_{Y,Z}\xi :=D_X\left( R^V_{Y,Z}\xi \right) -R^V_{D_XY,Z}\xi -R^V_{Y,D_X,Z}\xi -R^V_{Y,Z}\left( \nabla _X\xi \right) . \end{aligned}$$

$$\square $$

In the case $\phi \in C^{\infty }(M,P)$, we obtain

$$\begin{aligned} \left( \left[ D^*D,D\right] \phi \right) (X)=-D\phi \left( \mathrm {Ric}^M(X)\right) +R^P\left( D\phi (X),D\phi (e_i)\right) \left( D\phi (e_i)\right) \end{aligned}$$

and for $\xi \in \Gamma (T^*\Sigma ^{\otimes k}\otimes \phi ^*TP)$, we have

$$\begin{aligned}&\left( \left[ D^*D,D\right] \xi \right) (X,X_1,\ldots X_k)=2\sum _{l=1}^kD_{e_i}\xi \left( X_1,\ldots , R^M\left( e_i,X\right) X_l,\ldots X_k\right) \\&\quad +\sum _{l=1}^k\xi \left( X_1,\ldots ,D_{e_i}R^M(e_i,X)X_l,\ldots X_k\right) -D_{\mathrm {Ric}(X)}\xi (X_1,\ldots ,X_k)\\&\quad +D_{D\phi (e_i)}R^P(D\phi (X),D\phi (e_i))(\xi (X_1,\ldots X_k))\\&\quad +R^P\left( D^2\phi ({e_i,X}),D\phi (e_i)\right) (\xi ( X_1,\ldots X_k))\\&\quad +R^P\left( D\phi (X),D^2\phi (e_i,e_i)\right) \left( \xi (X_1,\ldots X_k)\right) \\&\quad +2R^P(D\phi (X),D\phi (e_i))\left( D_{e_i}\xi (X_1,\ldots ,X_k)\right) . \end{aligned}$$

Thus by iteration we get

$$\begin{aligned} \left[ D^*D,D^k\right] \phi&=\sum _{l=0}^{k-1}D^{l}R^M\star D^{k-l}\phi \\&\quad +\sum _{\sum l_i+\sum {m_j}=k-1}{}^{G}\nabla ^{l_1}R^P\star \underbrace{D^{m_1+1}\phi \star \cdots \star D^{m_{l_1}+1}\phi }_{l_1-\text {times}} \star D^{l_2+1}\phi \star D^{l_3+1}\phi \star D^{l_4+1}\phi . \end{aligned}$$

In the case of vector spinors, that is $\psi \in \Gamma (SM\otimes \phi ^*TP)$, we obtain

$$\begin{aligned} \left( \left[ D^*D,D\right] \psi \right) (X)&=-D_{\mathrm {Ric}(X)}\psi +2R^P(D\phi (X),D\phi (e_i))(D\psi (e_i))\\&\quad +2R^{SM}_{X,e_i}\cdot D_{e_i}\psi +D_{e_i}R^{SM}_{X,e_i}\cdot \psi \\&\quad +\nabla _{D\phi (e_i)}R^P(D\phi (X),D\phi (e_i))\psi + R^P\left( D^2\phi (e_i,X),D\phi (e_i)\right) \psi \\&\quad +R^P\left( D\phi (X),D^2\phi (e_i,e_i)\right) \psi \end{aligned}$$

and for $\xi \in \Gamma (T^*\Sigma ^{\otimes k}\otimes SM\otimes \phi ^*TP)$, we have

$$\begin{aligned} \left( \left[ D^*D,D\right] \xi \right) (X,X_1,\ldots ,X_k)&=-D_{\mathrm {Ric}(X)}\xi (X_1,\ldots ,X_k)\\&\quad +2R^P(D\phi (X),D\phi (e_i))\left( D_{e_i}\psi (X_1,\ldots ,X_k)\right) \\&\quad +2R^{SM}_{X,e_i}\cdot D_{e_i}\psi (X_1,\ldots ,X_k)+D_{e_i}R^{SM}_{X,e_i}\cdot \psi (X_1,\ldots ,X_k)\\&\quad +\nabla _{D\phi (e_i)}R^P(D\phi (X),D\phi (e_i))(\psi (X_1,\ldots ,X_k))\\&\quad + R^P\left( D^2\phi (e_i,X),D\phi (e_i)\right) (\psi (X_1,\ldots ,X_k))\\&\quad +R^P\left( D\phi (X),D^2\phi (e_i,e_i)\right) (\psi (X_1,\ldots ,X_k))\\&\quad +2\sum _{l=1}^kD_{e_i}\xi \left( X_1,\ldots , R^M(e_i,X)X_l,\ldots X_k\right) \\&\quad +\sum _{l=1}^k\xi \left( X_1,\ldots ,D_{e_i}R^M(e_i,X)X_l,\ldots X_k\right) . \end{aligned}$$

By iteration, we then obtain

$$\begin{aligned} \left[ D^*D,D^k\right] \psi&=\sum _{l=0}^{k-1}D^{l}R^M\star D^{k-l}\psi +\sum _{l=0}^{k}D^{l}R^{SM}\star D^{k-l}\psi \\&\quad +\sum _{\sum l_i+\sum {m_j}=k}{}^{G}\nabla ^{l_1}R^N\star \underbrace{D^{m_1+1}\phi \star \cdots \star D^{m_{l_1}+1}\phi }_{l_1-\text {times}}\star D^{l_2+1}\phi \star D^{l_3+1}\phi \star D^{l_4}\psi . \end{aligned}$$

We conclude this section with a very important lemma, which we will frequently make use of when deriving energy estimates.

#### Lemma 3.6

Let $(M^m,g)$ be an *m*-dimensional Riemannian manifold, *r* a natural number satisfying $r>\frac{m}{2}$, $E\rightarrow M$ a Riemannian vector bundle with a connection, $k\in \mathbb {N}$, $\xi _1,\ldots \xi _k\in H^r(E)$ and $l_i$ $i=1,\ldots k$ natural numbers satisfying $\sum _i l_i\le r$. Then the following inequality holds

$$\begin{aligned} \int _M \mathrm {I\!I}_{i=1}^k\left| \nabla ^{l_i}\xi _i\right| ^2\text { }dV\le C_{Sob}\cdot \mathrm {I\!I}_{i=1}^k\left\| \xi _i\right\| _{H^r}. \end{aligned}$$

If in addition, we have $\xi _{k+1}\in H^{r-1}(E)$ and $\sum _{i=1}^{k+1} l_i\le r-1$, then

$$\begin{aligned} \int _M \mathrm {I\!I}_{i=1}^{k+1}\left| \nabla ^{l_i}\xi _i\right| ^2\text { }dV\le C_{Sob}\cdot \mathrm {I\!I}_{i=1}^k\left\| \xi _i\right\| _{H^r}\cdot \left\| \xi _{k+1}\right\| _{H^{r-1}} . \end{aligned}$$

Here, $C_{sob}=C_{sob}(g,l_1,\ldots ,l_k,r)$ depends on the constant from the Sobolev embedding on $M$ and the numbers $l_1,\ldots ,l_k,r$.

#### Proof

We prove the first inequality, the second one is shown very similarly. Choose $p_i\in [1,\infty ]$, $i=1,\ldots ,l_k$ such that $\frac{1}{p_i}> \frac{1}{2}-\frac{r-l_i}{n}$ so that $\left\| \nabla ^{l_i}\xi _i\right\| _{L^{p_i}}\le C\left\| \xi _i\right\| _{H^r}$ by Sobolev embedding. Furthermore,

$$\begin{aligned} \sum _{i=1}^k\left( \frac{1}{2}-\frac{r-l_i}{m}\right)&=\frac{k}{2}-\frac{kr}{m}+\frac{1}{m}\left( \sum _{i=1}^kl_i\right) \\&=\frac{1}{2}+(k-1)\left( \frac{1}{2}-\frac{r}{m}\right) +\frac{1}{m}\left( \sum _{i=1}^kl_i-r\right) <\frac{1}{2} \end{aligned}$$

due to our assumptions. Therefore, we can choose the $p_i$ such that $\sum _{i=1}^k\frac{1}{p_i}=\frac{1}{2}$. An application of the Hölder inequality finishes the proof of the inequality. $\square$

### Energy of the map

We define the *k*th energy density of the map part as

3.3 $$\begin{aligned} e_k(\phi )=s^2\left| D^k\partial _t\phi \right| ^2+\left| D^kD\phi \right| ^2 \end{aligned}$$

and the *k*th energy as

3.4 $$\begin{aligned} E_k(\phi )=\int _\Sigma e_k(\phi )\text { }dV_{g_t}. \end{aligned}$$

The *k*th total energy is given by

3.5 $$\begin{aligned} F_k(\phi )=\sum _{l=0}^kE_l(\phi )=\int _\Sigma \sum _{l=0}^k e_l(\phi )\text { }dV_{g_t}=s^2\Vert \nabla _t\phi \Vert ^2_{H^k}+\Vert D\phi \Vert _{H^k}^2, \end{aligned}$$

where the Sobolev norms are taken with respect to the metric $g_t$. In (3.9) below, we also define the total energy $F_k(\psi )$ of the spinor part, which we already need in the following proposition:

#### Proposition 3.7

Let $T>0$, $r\in \mathbb {N}$, $r>(n-1)/2$, $k\in \left\{ 1,\ldots ,r\right\}$ and $(\phi ,\psi )$ be a solution of (2.4), (2.5) such that

$$\begin{aligned}&\phi \in C^0\left( [0,T),H^{r+1}(\Sigma ,P)\right) \cap C^1\left( [0,T),H^r(\Sigma ,P)\right) , \\&\psi \in C^0\left( [0,T),H^{r}\left( M,SM\otimes \phi ^*TP\right) \right) \cap C^1\left( [0,T),H^{r-1}\left( M,SM\otimes \phi ^*TP\right) \right) . \end{aligned}$$

Then the *k*th energy of the map satisfies the following inequality

3.6 $$\begin{aligned} \frac{d}{dt}E_k(\phi )&\le C_1(k)\left( \Vert \dot{g}\Vert _{C^{k-1}}+\Vert \partial _t\log N\Vert _{C^k}+s^{-1}\Vert D\log N\Vert _{C^k}+s^{-1}\Vert R^{\Sigma }\Vert _{C^{k-1}}\right) F_k(\phi )\nonumber \\&\quad -(n-2)s\dot{s}\int _{\Sigma }\left| D^k\nabla _t\phi \right| ^2\text { }dV_{g_t} \nonumber \\&\quad + C_2(k,n,g)s^{-1}\left\| R^P\right\| _{C^{k-1}}\sum _{l=1}^{k-1}F_r(\phi )^{2+l/2}\nonumber \\&\quad +C_3(k,n,g)s^{2-n}\left\| N^{2-n}\right\| _{C^k}\left\| R^P\right\| _{C^k}\Vert \partial _t\Vert _{C^k}\sum _{l=0}^k F_r(\phi )^{1+l/2}F_{r-1}(\psi )\nonumber \\&\quad +C_4(k,n,g)s^{1-n}\left\| N^{2-n}\right\| _{C^k}\left\| R^P\right\| _{C^k}\sum _{l=0}^k F_r(\phi )^{1+l/2}F_{r-1}(\psi )\nonumber \\&\quad + C_5(k,n,g)s^{3-2n}\left\| N^{4-2n}\right\| _{C^k}\left\| R^P\right\| _{C^{k+1}} \sum _{l=0}^k F_r(\phi )^{1/2+l/2}F_{r-1}(\psi )^2,\nonumber \\ \end{aligned}$$

where the positive constants $C_i$, $i\in \left\{ 1,\ldots ,5\right\}$ depend on *n*, *k* and the Sobolev constant of the metric $g_t$.

#### Proof

Assume for the moment that the initial data is compactly supported such that the solution is spacelike compactly supported. Using the general formula (3.3) we find

$$\begin{aligned} \frac{d}{dt}E_k(\phi )&=2\int _\Sigma \left\langle D^k\Box _h\phi ,D^k\nabla _t\phi \right\rangle \text { }dV_{g_t} +\frac{1}{2}\int _\Sigma \left( \left| D^kD\phi \right| -s^2\left| D^k\nabla _t\phi \right| ^2\right) {\text {tr}}_g\dot{g} \text { }dV_{g_t} \\&\quad +2s^2\int _\Sigma \left\langle \left[ \nabla _t,D^k\right] \nabla _t\phi ,D^k\nabla _t\phi \right\rangle \text { }dV_{g_t} +2\int _\Sigma \left\langle \left[ D^*D,D^k\right] \phi , D^k\nabla _t\phi \right\rangle \text { }dV_{g_t} \\&\quad +2\int _\Sigma \left\langle \left[ \nabla _t,D^{k+1}\right] \phi ,D^{k+1}\phi \right\rangle \text { }dV_{g_t}. \end{aligned}$$

Due to finite speed of propagation and an exhaustion procedure, this equality also holds generally for solutions in the above space. Note also that we will use Lemma 3.6 frequently in the proof without mentioning it explicitly. We have to estimate all terms on the right hand side and start by estimating the commutator terms

$$\begin{aligned}&s^2\int _\Sigma \left\langle D^k\nabla _t\phi ,\left[ \nabla _t,D^k\right] \nabla _t\phi \right\rangle \text { }dV_{g_t} \\&\quad =s^2\sum _{\sum l_i+\sum {m_j}=k-1}\int _\Sigma \nabla ^{l_1}R^P\star \underbrace{D^{m_1+1}\phi \star \cdots \star D^{m_{l_1}+1}\phi }_{l_1\text {{-}times}} \star D^{l_2}\partial _t\phi \star D^{l_3+1}\phi \star D^{l_4}\partial _t\phi \star D^k\partial _t\phi \text { }dV_{g_t}\\&\qquad +s^2\sum _{l=0}^{k-1}\int _\Sigma D^{k-l}\dot{g}\star D^{l+1}\partial _t\phi \star D^k\partial _t\phi \text { }dV_{g_t} \\&\quad \le C(k)s^2\left\| R^P\right\| _{C^{k-1}}\sum _{l=0}^{k-1}\Vert D\phi \Vert ^{l+1}_{H^r}\Vert \partial _t\phi \Vert ^3_{H^r} +C(k)s^2\Vert \dot{g}\Vert _{C^{k-1}}\Vert \partial _t\phi \Vert ^2_{H^k}\\&\quad \le C(k)s^{-1}\left\| R^P\right\| _{C^{k-1}}\sum _{l=0}^{k-1}\left( F_r(\phi )\right) ^{2+l/2} +C(k)\Vert \dot{g}\Vert _{C^{k-1}}F_k(\phi ). \end{aligned}$$

The second commutator can be controlled as follows

$$\begin{aligned}&\int _\Sigma \left\langle D^{k+1}\phi ,\left[ \nabla _t,D^{k+1}\phi \right] \right\rangle \text { }dV_{g_t} \\&\quad =\sum _{\sum l_i+\sum {m_j}=k-1}\int _\Sigma \nabla ^{l_1} R^P\star \underbrace{D^{m_1+1}\phi \star \cdots \star D^{m_{l_1}+1}\phi }_{l_1\text {{-}times}} \star D^{l_2}\partial _t\phi \star D^{l_3}D\phi \star D^{l_4}D\phi \star D^{k}D\phi \text { }dV_{g_t} \\&\qquad +\sum _{l=0}^{k}\int _\Sigma D^{k-l}\dot{g}\star D^{l}D\phi \star D^kD\phi \text { }dV_{g_t} \\&\quad \le C(k)\left\| R^P\right\| _{C^{k-1}}\sum _{l=0}^{k-1}\Vert D\phi \Vert _{H^r}^{l+3}\Vert \partial _t\phi \Vert _{H^r} +C(k)\Vert \dot{g}\Vert _{C^{k-1}}\Vert D\phi \Vert ^2_{H^k}\\&\quad \le C(k)s^{-1}\left\| R^P\right\| _{C^{k-1}}\sum _{l=0}^{k-1}(F_r(\phi ))^{2+l/2} +C(k)\Vert \dot{g}\Vert _{C^{k-1}}F_k(\phi ). \end{aligned}$$

The third commutator can be estimated as follows

$$\begin{aligned}&\int _\Sigma \left\langle D^k\partial _t\phi ,\left[ D^*D,D^k\right] \phi \right\rangle \text { }dV_{g_t} \\&\quad =\sum _{l=0}^{k-1}\int _\Sigma D^lR^{\Sigma }\star D^{k-l}\phi \star D^k\partial _t\phi \text { }dV_{g_t} \\&\qquad +\sum _{\sum l_i+\sum {m_j}=k-1}\int _\Sigma \nabla ^{l_1}R^P\star \underbrace{D^{m_1+1}\phi \star \cdots \star D^{m_{l_1}+1}\phi }_{l_1\text {{-}times}} \star D^{l_2}D\phi \star D^{l_3}D\phi \star D^{l_4}D\phi \star D^k\nabla _t\phi \text { }dV_{g_t} \\&\quad \le C(k)\left\| R^{\Sigma }\right\| _{C^{k-1}}\Vert D\phi \Vert _{H^{k-1}}\Vert \partial _t\phi \Vert _{H^k} +C(k)\left\| R^P\right\| _{C^{k-1}}\sum _{l=0}^{k-1}\Vert D\phi \Vert ^{l+3}_{H^r}\Vert \partial _t\phi \Vert _{H^r}\\&\quad \le C(k)s^{-1}\left\| R^{\Sigma }\right\| _{C^{k-1}}F_k(\phi )+C(k)s^{-1}\left\| R^P\right\| _{C^{k-1}}\sum _{l=0}^{k-1}(F_r(\phi ))^{2+l/2}. \end{aligned}$$

As a second step we estimate the terms that arise when inserting the equation (2.4) for $\phi$ into (3.3). In order to estimate the first term we calculate

$$\begin{aligned} (Ns)^{-1}\nabla _{\nabla (Ns)}\phi =-s\dot{s}\nabla _t\phi -s^2N^{-1}\partial _tN\nabla _t\phi +N^{-1}\left\langle DN, D\phi \right\rangle _g. \end{aligned}$$

This allows us to derive the following estimate

$$\begin{aligned}&\int _{\Sigma }\left\langle D^k\left( (Ns)^{-1}\nabla _{\nabla (Ns)}\phi \right) ,D^k\nabla _t\phi \right\rangle \text { }dV_{g_t} \\&\quad =-s\dot{s}\int _{\Sigma }\left| D^k\nabla _t\phi \right| ^2\text { }dV_{g_t} +\sum _{l=0}^k\int _{\Sigma } D^{l}\left( \frac{DN}{N}\right) \star D^{k-l}D\phi \star D^k\nabla _t\phi \text { }dV_{g_t} \\&\qquad +s^2\sum _{l_i}^k\int _{\Sigma } D^{l_1}\left( \frac{\partial _tN}{N}\right) \star D^{l_2}\nabla _t\phi \star D^k\nabla _t\phi \text { }dV_{g_t} \\&\quad \le -s\dot{s}\int _{\Sigma }\left| D^k\nabla _t\phi \right| ^2\text { }dV_{g_t}+C(k)s^{-1}\Vert D\log N\Vert _{C^k}F_k(\phi ) +C(k)\Vert \partial _t\log N\Vert _{C^k}F_k(\phi ). \end{aligned}$$

In order to treat the second term on the right hand side of (2.4) we have to consider spatial and time derivatives separately

$$\begin{aligned}&D^k\left( R^P(\psi ,i\partial _t\cdot \psi )d\phi (\partial _t)\right) \\&\quad =\sum _{\sum l_i+\sum {m_j}=k} \nabla ^{l_1}R^P\star \underbrace{D^{m_1+1}\phi \star \cdots \star D^{m_{l_1}+1}\phi }_{l_1\text {{-}times}}\star D^{l_2}\partial _t\star D^{l_3}\psi \star D^{l_4}\psi \star D^{l_5}\nabla _t\phi , \\&D^k\left( R^P(\psi ,i\partial _i\cdot \psi )d\phi (\partial _i)\right) \\&\quad =\sum _{\sum l_i+\sum {m_j}=k} \nabla ^{l_1}R^P\star \underbrace{D^{m_1+1}\phi \star \cdots \star D^{m_{l_1}+1}\phi }_{l_1\text {{-}times}}\star D^{l_2}\psi \star D^{l_3}\psi \star D^{l_4}D\phi , \end{aligned}$$

such that we find

$$\begin{aligned}&D^k h^{\alpha \beta }\left( N^{2-n}R^P(\psi ,i\partial _\alpha \cdot \psi )d\phi (\partial _\beta )\right) \nonumber \\&\quad =\sum _{\sum l_i+\sum {m_j}=k} D^{l_1}N^{2-n}\star \left( s^2\nabla ^{l_2}R^P\star \underbrace{D^{m_1+1}\phi \star \cdots \star D^{m_{l_2}+1}\phi }_{l_2\text {{-}times}} \star D^{l_3}\partial _t\star D^{l_4}\psi \star D^{l_5}\psi \star D^{l_6}\nabla _t\phi \right. \nonumber \\&\left. \qquad +\nabla ^{l_2}R^P\star \underbrace{D^{m_1+1}\phi \star \cdots \star D^{m_{l_2}+1}\phi }_{l_2\text {{-}times}} \star D^{l_3}\psi \star D^{l_4}\psi \star D^{l_5}D\phi \right) . \end{aligned}$$

This allows us to derive the following estimate:

$$\begin{aligned}&\int _{\Sigma }\left\langle D^k\left( -\frac{1}{2}h^{\alpha \beta }(Ns)^{2-n}R^P(\psi ,i\partial _\alpha \cdot \psi )d\phi (\partial _\beta )\right) ,D^k\nabla _t\phi \right\rangle \text { }dV_{g_t}\\&\quad =-\frac{s^{2-n}}{2}\sum _{\sum l_i+\sum {m_j}=k}\int _{\Sigma }D^{l_1}N^{2-n} \\&\qquad \star s^2\left( \nabla ^{l_2}R^P\star \underbrace{D^{m_1+1}\phi \star \cdots \star D^{m_{l_2}+1}\phi }_{l_2\text {{-}times}} \star D^{l_3}\partial _t\star D^{l_4}\psi \star D^{l_5}\psi \star D^{l_6}\nabla _t\phi \star D^k\nabla _t\phi \right. \\&\left. \qquad +D^{l_2}R^P\star \underbrace{D^{m_1+1}\phi \star \cdots \star D^{m_{l_2}+1}\phi }_{l_2\text {{-}times}} \star D^{l_3}\psi \star D^{l_4}\psi \star D^{l_5}D\phi \star D^k\nabla _t\phi \right) \text { }dV_{g_t}\\&\quad \le C(k,n)s^{4-n}\left\| N^{2-n}\right\| _{C^k}\left\| R^P\right\| _{C^k}\Vert \partial _t\Vert _{C^k}\sum _{l=0}^k\Vert D\phi \Vert ^l_{H^r}\Vert \psi \Vert ^2_{H^r}\Vert \nabla _t\phi \Vert ^2_{H^r} \\&\qquad +C(k,n)s^{2-n}\left\| N^{2-n}\right\| _{C^k}\left\| R^P\right\| _{C^k}\sum _{l=0}^k\Vert D\phi \Vert ^{l+1}_{H^r}\Vert \psi \Vert ^2_{H^r}\Vert \nabla _t\phi \Vert _{H^r}\\&\quad \le C(k,n)s^{2-n}\left\| N^{2-n}\right\| _{C^k}\left\| R^P\right\| _{C^k}\Vert \partial _t\Vert _{C^k}\sum _{l=0}^k F_r(\phi )^{1+l/2}F_{r-1}(\psi )\\&\qquad +C(k,n)s^{1-n}\left\| N^{2-n}\right\| _{C^k}\left\| R^P\right\| _{C^k}\sum _{l=0}^k F_r(\phi )^{1+l/2}F_{r-1}(\psi ). \end{aligned}$$

The third term on the right hand side of (2.4) can be computed as

$$\begin{aligned}&D^k\left( N^{4-2n}\left\langle \left( \nabla R^P\right) ^\sharp (\psi ,\psi )\psi ,\psi \right\rangle \right) \\&\quad =\sum _{\sum l_i+\sum {m_j}=k} D^{l_1}N^{4-2n}\star \nabla ^{l_2+1}R^P\star \underbrace{D^{m_1+1}\phi \star \cdots \star D^{m_{l_2}+1}\phi }_{l_2\text {{-}times}}\star D^{l_3}\psi \star D^{l_4}\psi \star D^{l_5}\psi \star D^{l_6}\psi . \end{aligned}$$

This allows us to derive the following estimate

$$\begin{aligned}&\int _{\Sigma }\left\langle D^k\left( (Ns)^{4-2n}\left\langle \left( \nabla R^P\right) ^\sharp (\psi ,\psi )\psi ,\psi \right\rangle \right) ,D^k\partial _t\phi \right\rangle \text { }dV_{g_t}\\&\quad =s^{4-2n}\sum _{\sum l_i+\sum {m_j}=k}\int _\Sigma D^{l_1}N^{4-2n}\star \nabla ^{l_2+1}R^P\\&\qquad \star \underbrace{D^{m_1+1}\phi \star \cdots \star D^{m_{l_2}+1}\phi }_{l_2\text {{-}times}}\star D^{l_3}\psi \star D^{l_4}\psi \star D^{l_5}\psi \star D^{l_6}\psi \star D^{k}\partial _t\phi \text { }dV_{g_t} \\&\quad \le s^{4-2n}C(k)\left\| N^{4-2n}\right\| _{C^k}\left\| R^P\right\| _{C^{k+1}}\sum _{l=0}^k\Vert D\phi \Vert ^l_{H^r}\Vert \psi \Vert ^4_{H^r}\Vert \partial _t\phi \Vert _{H^r}\\&\quad \le s^{3-2n}C(k)\left\| N^{4-2n}\right\| _{C^k}\left\| R^P\right\| _{C^{k+1}} \sum _{l=0}^k F_r(\phi )^{1/2+l/2}F_{r-1}(\psi )^2. \end{aligned}$$

Adding up the different contributions concludes the proof. $\square$

### Energy of the spinor

We define the *k*th energy density of the spinor part as

3.7 $$\begin{aligned} e_k(\psi )=s^2\left| D^k\nabla _t\psi \right| ^2+\left| D^kD\psi \right| ^2 \end{aligned}$$

and the *k*th energy as

3.8 $$\begin{aligned} E_k(\psi )=\int _\Sigma e_k(\psi )\text { }dV_{g_t}. \end{aligned}$$

The *k*th total energy is given by

3.9 $$\begin{aligned} F_k(\psi )=\sum _{l=0}^kE_l(\psi )+\Vert \psi \Vert _{L^2}^2=\int _\Sigma \sum _{l=0}^k e_l(\psi )\text { }dV_{g_t}+\Vert \psi \Vert _{L^2}^2=s^2\Vert \nabla _t\psi \Vert _{H^k}^2+\Vert \psi \Vert _{H^{k+1}}^2. \end{aligned}$$

Note that

3.10 $$\begin{aligned} \frac{d}{dt}\Vert \psi \Vert _{L^2}^2\le 2\Vert \nabla _t\psi \Vert _{L^2}\Vert \psi \Vert _{L^2}+\frac{1}{2}\Vert {\text {tr}}{\dot{g}}\Vert _{L^{\infty }}\Vert \psi \Vert _{L^2}^2\le \left( s^{-1}+\frac{1}{2}\Vert {\text {tr}}{\dot{g}}\Vert _{L^{\infty }}\right) F_1(\psi ). \end{aligned}$$

#### Proposition 3.8

Let $T>0$, $r\in \mathbb {N}$, $r>(n-1)/2$, $k\in \left\{ 1,\ldots ,r-1\right\}$ and $(\phi ,\psi )$ be a solution of (2.4), (2.5) such that

$$\begin{aligned}&\phi \in C^0\left( [0,T),H^{r+1}(\Sigma ,P)\right) \cap C^1\left( [0,T),H^r(\Sigma ,P)\right) , \\&\psi \in C^0\left( [0,T),H^{r}\left( M,SM\otimes \phi ^*TP\right) \right) \cap C^1\left( [0,T),H^{r-1}\left( M,SM\otimes \phi ^*TP\right) \right) . \end{aligned}$$

Then the $k$th energy of the spinor satisfies the following inequality

3.11 $$\begin{aligned} \begin{aligned} \frac{d}{dt}E_k(\psi )&\le C_1(k,n)\left( \left\| R^{SM}(\partial _t,\cdot )\right\| _{C^{k-1}}+\Vert \dot{g}\Vert _{C^k}+\Vert D^*e_0\Vert _{L^\infty }\right) F_k(\psi )\\&\quad + C_2(n,k)\cdot s^{-1}\left( \left\| R^{\Sigma }\right\| _{C^{k-1}}+\left\| R^{SM}\right\| _{C^k}+\left\| \mathrm {scal}^M\right\| _{C^k}\right) F_k(\psi )\\&\quad + C_3(n,k,g)\left( s^{-1}+\Vert \partial _t\Vert _{C^k}\right) \left\| R^P\right\| _{C^{k+1}}\sum _{l=0}^kF_r(\phi )^{l/2+1}F_{r-1}(\psi )\\&\quad + C_4(n,k,g)s^{2-n}\left\| N^{2-n}\right\| _{C^k}\left\| R^P\right\| _{C^{k+1}}\Vert \partial _t\Vert _{C^k}\sum _{l=0}^{k+1}F_r(\phi )^{l/2}F_{r-1}(\psi )^2\\&\quad + (n-2)\cdot C_5(n,k,g)s^{3-n}\left\| N^{2-n}\partial _t\log N\right\| _{C^k}\left\| R^P\right\| _{C^{k}}\Vert \partial _t\Vert _{C^k}\sum _{l=0}^{k}F_r(\phi )^{l/2}F_{r-1}(\psi )^2\\&\quad + (n-2)\cdot C_6(n,k,g){\dot{s}}s^{2-n}\left\| N^{2-n}\right\| _{C^k}\left\| R^P\right\| _{C^{k}}\Vert \partial _t\Vert _{C^k}\sum _{l=0}^{k}F_r(\phi )^{l/2}F_{r-1}(\psi )^2\\&\quad + C_7(n,k,g)s^{1-n}\left\| N^{2-n}\right\| _{C^{k+1}}\left\| R^P\right\| _{C^{k+1}}\sum _{l=0}^{k+1}F_r(\phi )^{l/2}F_{r-1}(\psi )^2\\&\quad + C_8(n,k,g)s^{1-n}\left\| N^{2-n}\right\| _{C^{k}}\left\| R^P\right\| _{C^{k}}^2\sum _{l=0}^{2k}F_r(\phi )^{l/2}F_{r-1}(\psi )^3, \end{aligned} \end{aligned}$$

where the positive constants $C_i$, $i\in \left\{ 3,\ldots ,8\right\}$ depend on *n*, *k* and the Sobolev constant of the metric $g_t$.

#### Remark 3.9

The curvature quantities coming from the spinor bundle above have to be understood as the following sections: $R^{SM}(\partial _t,\cdot )\in \Gamma (T^*\Sigma \otimes \mathrm {End}(SM))$, $R^{SM}=R^{SM}(.,.)\in \Gamma (\Lambda ^2T\Sigma \otimes \mathrm {End}(SM))$, both by restricting to vectors tangent to $\Sigma$. Moreover $\partial _t\in \Gamma (\mathrm {End}(SM))$ acts by Clifford multiplication. The $C^k$ norms with respect to $g_t$ are then defined in a canonical way.

#### Proof

In order to derive an energy estimate for the spinor we make use of (3.2). As before, we may at first assume that the solution is spacelike compactly supported before we obtain this equality for the general case by an exhaustion procedure. We will also frequently use Lemma 3.6 in this proof. Again, we have to estimate all terms on the right hand side and start by estimating the commutator terms:

$$\begin{aligned}&s^2\int _\Sigma \left\langle D^k\nabla _t\psi ,\left[ \nabla _t,D^k\right] \psi \right\rangle \text { }dV_{g_t} \\&\quad =s^2\sum _{l=0}^{k-1}\int _\Sigma D^l\left( R^{SM}(\partial _t,\cdot )\right) \star D^{k-l-1}\nabla _t\psi \star D^k\nabla _t\psi \text { }dV_{g_t} \\&\qquad +s^2\sum _{l=0}^{k-1}\int _\Sigma D^{k-l}\dot{g}\star D^{l+1}\nabla _t\psi \star D^k\nabla _t\psi \text { }dV_{g_t}\\&\qquad +s^2\sum _{\sum l_i+\sum {m_j}=k-1}\int _\Sigma \nabla ^{l_1} R^P\\&\qquad \star \underbrace{D^{m_1+1}\phi \star \cdots \star D^{m_{l_1}+1}\phi }_{l_1\text {{-}times}} \star D^{l_2}\nabla _t\phi \star D^{l_3}D\phi \star D^{l_4}\nabla _t\psi \star D^k\nabla _t\psi \text { }dV_{g_t}\\&\quad \le s^2 C(k)\left\| R^{SM}(\partial _t,\cdot )\right\| _{C^{k-1}}\Vert \nabla _t\psi \Vert ^2_{H^k} +s^2C(k)\Vert \dot{g}\Vert _{C^k}\Vert \nabla _t\psi \Vert ^2_{H^k} \\&\qquad +s^2C(k)\left\| R^P\right\| _{C^{k-1}}\sum _{l=0}^{k-1}\Vert D\phi \Vert _{H^r}^{l+1}\Vert \partial _t\phi \Vert _{H^r}\Vert \nabla _t\psi \Vert ^2_{H^{r-1}}\\&\quad \le C(k)\left\| R^{SM}(\partial _t,\cdot )\right\| _{C^{k-1}} F_k(\psi )+ C(k)\Vert \dot{g}\Vert _{C^k} F_k(\psi )\\&\qquad +s^{-1}C(k)\left\| R^P\right\| _{C^{k-1}}\sum _{l=0}^{k-1}F_r(\phi )^{l/2+1}F_{r-1}(\psi ). \end{aligned}$$

The second commutator term can be estimated as follows:

$$\begin{aligned}&\int _\Sigma \left\langle \left[ D^*D,D^k\right] \psi , D^k\nabla _t\psi \right\rangle \text { }dV_{g_t} \\&\quad =\sum _{l=0}^{k-1}\int _\Sigma D^lR^M\star D^{k-l}\psi \star D^k\nabla _t\psi \text { }dV_{g_t} +\sum _{l=0}^k\int _\Sigma D^l R^{SM}\star D^{k-l}\psi \star D^k\nabla _t\psi \text { }dV_{g_t} \\&\qquad +\sum _{\sum l_i+\sum {m_j}=k}\int _\Sigma \nabla ^{l_1} R^P\star \underbrace{D^{m_1+1}\phi \star \cdots \star D^{m_{l_1}+1}\phi }_{l_1\text {{-}times}} \star D^{l_2}D\phi \star D^{l_3}D\phi \star D^{l_4}\psi \star D^k\nabla _t\psi \text { }dV_{g_t} \\&\quad \le C(k)\left\| R^M\right\| _{C^{k-1}}\Vert \psi \Vert _{H^k}\Vert \nabla _t\psi \Vert _{H^k} +C(k)\left\| R^{SM}\right\| _{C^{k}}\Vert \psi \Vert _{H^{k}}\Vert \nabla _t\psi \Vert _{H^k} \\&\qquad +C(k)\left\| R^P\right\| _{C^{k}}\sum _{l=0}^{k}\Vert D\phi \Vert _{H^r}^{l+2}\Vert \psi \Vert _{H^r}\Vert \nabla _t\psi \Vert _{H^{r-1}}\\&\quad \le s^{-1}C(k)\left( \left\| R^M\right\| _{C^{k-1}}+\left\| R^{SM}\right\| _{C^{k}}\right) F_k(\psi )\\&\qquad +s^{-1}C(k)\left\| R^P\right\| _{C^{k}}\sum _{l=0}^{k}F_r(\phi )^{l/2+1}F_{r-1}(\psi ). \end{aligned}$$

The third commutator can be controlled as follows:

$$\begin{aligned}&\int _\Sigma \left\langle \left[ \nabla _t,D^{k+1}\right] \psi ,D^{k+1}\psi \right\rangle \text { }dV_{g_t}\\&\quad =\sum _{l=0}^k\int _\Sigma D^l\left( R^{SM}(\partial _t,\cdot )\right) \star D^{k-l}\psi \star D^{k+1}\psi \text { }dV_{g_t}\\&\qquad +\sum _{l=0}^k\int _\Sigma D^{k-l-1}\dot{g}\star D^{l+1}\psi \star D^{k+1}\psi \text { }dV_{g_t} \\&\qquad +\sum _{\sum l_i+\sum {m_j}=k-1}\int _\Sigma \nabla ^{l_1} R^P\star \underbrace{D^{m_1+1}\phi \star \cdots \star D^{m_{l_1}+1}\phi }_{l_1\text {{-}times}}\\&\qquad \star D^{l_2}\partial _t\phi \star D^{l_3}D\phi \star D^{l_4}\psi \star D^{k+1}\psi \text { }dV_{g_t} \\&\quad \le C(k)\left\| R^{SM}(\partial _t,\cdot )\right\| _{C^k}\Vert \psi \Vert ^2_{H^{k+1}}+C(k)\Vert \dot{g}\Vert _{C^k}\Vert \psi \Vert ^2_{H^{k+1}} \\&\qquad +C(k)\left\| R^P\right\| _{C^k}\sum _{l=0}^{k}\Vert D\phi \Vert _{H^r}^{l+1}\Vert \nabla _t\phi \Vert _{H^r}\Vert \psi \Vert ^2_{H^{r}}\\&\quad \le C(k)\left\| R^{SM}(\partial _t,\cdot )\right\| _{C^k}F_k(\psi )+ C(k)\Vert \dot{g}\Vert _{C^k}F_k(\psi )\\&\qquad +s^{-1}C(k)\left\| R^P\right\| _{C^k}\sum _{l=0}^{k}F_r(\phi )^{l/2+1}F_{r-1}(\psi ). \end{aligned}$$

As a next step we estimate the terms that arise when inserting (2.5) into (3.3). The first term can easily be controlled as

$$\begin{aligned} \int _\Sigma \left\langle D^k\left( \frac{\mathrm {scal}^M}{4}\psi \right) ,D^k\nabla _t\psi \right\rangle \text { }dV_{g_t}&=\frac{1}{4}\sum _{l=0}^k\int _\Sigma D^{l}\mathrm {scal}^M\star D^{k-l}\psi \star D^k\nabla _t\psi \text { }dV_{g_t} \\&\le C(k)\left\| \mathrm {scal}^M\right\| _{C^k}s^{-1}F_k(\psi ). \end{aligned}$$

Regarding the second term on the right hand side of (2.5) we again expand space and time contributions

$$\begin{aligned} h^{\alpha \gamma }h^{\beta \delta }\partial _\alpha \cdot \partial _\beta \cdot R^P\left( d\phi \left( \partial _\gamma \right) ,d\phi (\partial _\delta )\right) \psi&=-2s^2g^{ij}\partial _t\cdot \partial _i\cdot R^P\left( d\phi (\partial _t),d\phi (\partial _j)\right) \psi \\&\quad +g^{ij}g^{kl}\partial _i\cdot \partial _k\cdot R^P\left( d\phi (\partial _j),d\phi (\partial _l)\right) \psi . \end{aligned}$$

Note that we do not get a term proportional to $|d\phi (\partial _t)|^2$ due to symmetry reasons. We then find

$$\begin{aligned}&D^k\left( h^{\alpha \gamma }h^{\beta \delta }\partial _\alpha \cdot \partial _\beta \cdot R^P\left( d\phi (\partial _\gamma ),d\phi (\partial _\delta )\right) \psi \right) \\&\quad =\sum _{\sum l_i+\sum {m_j}=k}\left( D^{l_1}R^P\star \underbrace{D^{m_1+1}\phi \star \cdots \star D^{m_{l_1}+1}\phi }_{l_1\text {{-}times}} \star D^{l_2}D\phi \star D^{l_3}D\phi \star D^{l_4}\psi \right) \\&\qquad +s^2\sum _{\sum l_i+\sum {m_j}=k}\left( D^{l_1}\partial _t\star D^{l_2}R^P\star \underbrace{D^{m_1+1}\phi \star \cdots \star D^{m_{l_2}+1}\phi }_{l_2\text {{-}times}} \star D^{l_3}d\phi (\partial _t)\star D^{l_4}d\phi \star D^{l_5}\psi \right) . \end{aligned}$$

This allows us to derive the following estimate

$$\begin{aligned}&\int _\Sigma \left\langle D^k\left( h^{\alpha \gamma }h^{\beta \delta }\partial _\alpha \cdot \partial _\beta \cdot R^P\left( d\phi (\partial _\gamma ),d\phi (\partial _\delta )\right) \psi \right) ,D^k\nabla _t\psi \right\rangle \text { }dV_{g_t}\\&\quad =\sum _{\sum l_i+\sum {m_j}=k}\int _\Sigma D^{l_1}R^P\star \underbrace{D^{m_1+1}\phi \star \cdots \star D^{m_{l_1}+1}\phi }_{l_1\text {{-}times}} \star D^{l_2}d\phi \star D^{l_3}d\phi \star D^{l_4}\psi \star D^k\nabla _t\psi \text { }dV_{g_t} \\&\qquad +s^2\sum _{\sum l_i+\sum {m_j}=k}\int _\Sigma D^{l_1}\partial _t\star D^{l_2}R^P\star \underbrace{D^{m_1+1}\phi \star \cdots \star D^{m_{l_2}+1}\phi }_{l_2\text {{-}times}} \\&\qquad \star D^{l_3}d\phi (\partial _t)\star D^{l_4}d\phi \star D^{l_5}\psi \star D^k\nabla _t\psi \text { }dV_{g_t} \\&\quad \le C(k)\left\| R^P\right\| _{C^k}\sum _{l=0}^k\Vert D\phi \Vert _{H^r}^{l+2}\Vert \psi \Vert _{H^{r}}\Vert \nabla _t\psi \Vert _{H^{k}}\\&\qquad +s^2C(k)\Vert \partial _t\Vert _{C^k}\left\| R^P\right\| _{C^k}\sum _{l=0}^k\Vert D\phi \Vert _{H^r}^{l+1}\Vert \nabla _t\phi \Vert _{H^r}\Vert \psi \Vert _{H^r}\Vert \nabla _t\psi \Vert _{H^{k}}\\&\quad \le s^{-1}C(k)\left\| R^P\right\| _{C^k}\sum _{l=0}^k F_r(\phi )^{l/2+1}F_{r-1}(\psi ){+}C(k)\Vert \partial _t\Vert _{C^k}\left\| R^P\right\| _{C^k}\sum _{l=0}^k F_r(\phi )^{l/2+1}F_{r-1}(\psi ). \end{aligned}$$

To manipulate the third term on the right hand side of (2.5) we first consider the derivative with respect to $t$. We find

$$\begin{aligned} \nabla _{t}\left( R^P(\psi ,\psi )(Ns)^{2-n}\right) \partial _t\cdot \psi&=\left( \nabla _{d\phi (\partial _t)}R^P\right) (\psi ,\psi )(Ns)^{2-n}\partial _t\cdot \psi \\&\quad +2R^P\left( \nabla _t\psi ,\psi \right) (Ns)^{2-n}\partial _t\cdot \psi \\&\quad +(2-n)R^P(\psi ,\psi )(Ns)^{2-n}\partial _t\log N\partial _t\cdot \psi \\&\quad +(2-n)\dot{s}s^{1-n}N^{2-n}R^P(\psi ,\psi )\partial _t\cdot \psi . \end{aligned}$$

As a next step we calculate the $k$-th spatial derivative of this expression

$$\begin{aligned}&D^k\left( \left( \nabla _{d\phi (\partial _t)}R^P\right) (\psi ,\psi )N^{2-n}\partial _t\cdot \psi \right) =\sum _{\sum l_i+\sum {m_j}=k}^kD^{l_1}N^{2-n}\star D^{l_2+1}R^P\\&\quad \star \underbrace{D^{m_1+1}\phi \star \cdots \star D^{m_{l_2+1}+1}\phi }_{l_2+1\text {{-}times}} \star D^{l_3}\psi \star D^{l_4}\psi \star D^{l_5}\partial _t\star D^{l_6}\psi \star D^{l_7}d\phi (\partial _t),\\&D^k\left( R^P(\nabla _t\psi ,\psi )N^{2-n}\partial _t\cdot \psi \right) =\sum _{\sum l_i+\sum {m_j}=k}^kD^{l_1}N^{2-n}\star D^{l_2}R^P\\&\quad \star \underbrace{D^{m_1+1}\phi \star \cdots \star D^{m_{l_2}+1}\phi }_{l_2\text {{-}times}} \star D^{l_3}\nabla _t\psi \star D^{l_4}\psi \star D^{l_5}\partial _t\star D^{l_6}\psi ,\\&D^k\left( R^P(\psi ,\psi )N^{2-n}\partial _t\log N\partial _t\cdot \psi \right) =\sum _{\sum l_i+\sum {m_j}=k}^kD^{l_1}N^{2-n}\partial _t\log N\star D^{l_2}R^P\\&\quad \star \underbrace{D^{m_1+1}\phi \star \cdots \star D^{m_{l_2}+1}\phi }_{l_2\text {{-}times}} \star D^{l_3}\psi \star D^{l_4}\psi \star D^{l_5}\partial _t\star D^{l_6}\psi ,\\&D^k\left( N^{2-n}R^P(\psi ,\psi )\partial _t\cdot \psi \right) =\sum _{\sum l_i+\sum {m_j}=k}^kD^{l_1}N^{2-n}\star D^{l_2}R^P\\&\quad \star \underbrace{D^{m_1+1}\phi \star \cdots \star D^{m_{l_2}+1}\phi }_{l_2\text {{-}times}} \star D^{l_3}\psi \star D^{l_4}\psi \star D^{l_5}\partial _t\star D^{l_6}\psi . \end{aligned}$$

These manipulations allow us to derive the following estimates:

$$\begin{aligned}&s^2\int _\Sigma \left\langle D^k\left( \nabla _{t}\left( R^P(\psi ,\psi )(Ns)^{2-n}\right) \partial _t\cdot \psi \right) ,D^k\nabla _t\psi \right\rangle \text { }dV_{g_t} \\&\quad =s^{4-n}\sum _{\sum l_i+\sum {m_j}=k}\int _\Sigma D^{l_1}N^{2-n}\star D^{l_2+1}R^P\star \underbrace{D^{m_1+1}\phi \star \cdots \star D^{m_{l_2+1}+1}\phi }_{l_2+1\text {{-}times}}\\&\quad \qquad \star D^{l_3}\psi \star D^{l_4}\psi \star D^{l_5}\partial _t\star D^{l_6}\psi \star D^{l_7}d\phi (\partial _t)\star D^k\nabla _t\psi \text { }dV_{g_t} \\&\qquad +s^{4-n}\sum _{\sum l_i+\sum {m_j}=k}\int _\Sigma D^{l_1}N^{2-n}\star D^{l_2}R^P\star \underbrace{D^{m_1+1}\phi \star \cdots \star D^{m_{l_2}+1}\phi }_{l_2\text {{-}times}}\\&\quad \qquad \star D^{l_3}\nabla _t\psi \star D^{l_4}\psi \star D^{l_5}\partial _t\star D^{l_6}\psi \star D^k\nabla _t\psi \text { }dV_{g_t} \\&\qquad +(2-n)\dot{s}s^{3-n}\sum _{\sum l_i+\sum {m_j}=k}\int _\Sigma D^{l_1}N^{2-n}\star D^{l_2}R^P\star \underbrace{D^{m_1+1}\phi \star \cdots \star D^{m_{l_2}+1}\phi }_{l_2\text {{-}times}}\\&\quad \qquad \star D^{l_3}\psi \star D^{l_4}\psi \star D^{l_5}\partial _t\star D^{l_6}\psi \star D^k\nabla _t\psi \text { }dV_{g_t} \\&\qquad +(2-n)s^{4-n}\sum _{\sum l_i+\sum {m_j}=k}\int _\Sigma D^{l_1}N^{2-n}\partial _t\log N\star D^{l_2}R^P\star \underbrace{D^{m_1+1}\phi \star \cdots \star D^{m_{l_2}+1}\phi }_{l_2\text {{-}times}}\\&\quad \qquad \star D^{l_3}\psi \star D^{l_4}\psi \star D^{l_5}\partial _t\star D^{l_6}\psi \star D^k\nabla _t\psi \text { }dV_{g_t} \\&\quad \le C(k)s^{4-n}\left\| N^{2-n}\right\| _{C^k}\left\| R^P\right\| _{C^{k+1}}\Vert \partial _t\Vert _{C^k}\sum _{l=0}^k\Vert D\phi \Vert ^l_{H^r}\Vert \psi \Vert ^3_{H^r}\Vert \nabla _t\phi \Vert _{H^r}\Vert \nabla _t\psi \Vert _{H^k} \\&\qquad +C(k)s^{4-n}\left\| N^{2-n}\right\| _{C^k}\left\| R^P\right\| _{C^{k}}\Vert \partial _t\Vert _{C^k}\sum _{l=0}^k\Vert D\phi \Vert ^l_{H^r}\Vert \psi \Vert ^2_{H^r}\Vert \nabla _t\psi \Vert ^2_{H^k} \\&\qquad +C(k,n)(n-2)\dot{s}s^{3-n}\left\| N^{2-n}\right\| _{C^k}\left\| R^P\right\| _{C^{k}}\Vert \partial _t\Vert _{C^k}\sum _{l=0}^k\Vert D\phi \Vert ^l_{H^r}\Vert \psi \Vert ^3_{H^r}\Vert \nabla _t\psi \Vert _{H^k}\\&\qquad +C(k,n)(n-2)s^{4-n}\left\| N^{2-n}\partial _t\log N\right\| _{C^k}\left\| R^P\right\| _{C^{k}}\Vert \partial _t\Vert _{C^k}\sum _{l=0}^k\Vert D\phi \Vert ^l_{H^r}\Vert \psi \Vert ^3_{H^r}\Vert \nabla _t\psi \Vert _{H^k}\\&\quad \le C(k)s^{2-n}\left\| N^{2-n}\right\| _{C^k}\left\| R^P\right\| _{C^{k+1}}\Vert \partial _t\Vert _{C^k}\sum _{l=0}^kF_r(\phi )^{l/2+1/2}F_{r-1}(\psi )^2\\&\qquad +C(k)s^{2-n}\left\| N^{2-n}\right\| _{C^k}\left\| R^P\right\| _{C^{k}}\Vert \partial _t\Vert _{C^k} \sum _{l=0}^kF_r(\phi )^{l/2}F_{r-1}(\psi )^2\\&\qquad +C(k,n)(n{-}2)\dot{s}s^{2-n}\left\| N^{2-n}\right\| _{C^k}\left\| R^P\right\| _{C^{k}}\Vert \partial _t\Vert _{C^k}\sum _{l=0}^kF_r(\phi )^{l/2}F_{r-1}(\psi )^2\\&\qquad +C(k,n)(n-2)s^{3-n}\left\| N^{2-n}\partial _t\log N\right\| _{C^k}\left\| R^P\right\| _{C^{k}}\Vert \partial _t\Vert _{C^k}\sum _{l=0}^kF_r(\phi )^{l/2}F_{r-1}(\psi )^2. \end{aligned}$$

As a next step we take care of the spatial derivatives in the third term on the right hand side of (2.5). These can be computed as

$$\begin{aligned} D\left( R^P(\psi ,\psi )(Ns)^{2-n}\right) \cdot \psi&=\left( D_{d\phi }R^P\right) (\psi ,\psi )(Ns)^{2-n}\cdot \psi +2R^P(D\psi ,\psi )(Ns)^{2-n}\cdot \psi \\&\quad +s^{2-n}R^P(\psi ,\psi )D(N)^{2-n}\cdot \psi . \end{aligned}$$

The $k$th spatial derivative of these terms acquire the forms

$$\begin{aligned}&D^k\big (\left( D_{d\phi }R^P\right) (\psi ,\psi )(Ns)^{2-n}\cdot \psi \big )\\&\quad =s^{2-n}\sum _{\sum l_i+\sum {m_j}=k} D^{l_1+1}R^P\star \underbrace{D^{m_1+1}\phi \star \cdots \star D^{m_{l_1+1}+1}\phi }_{(l_1+1)\text {{-}times}} \star D^{l_2}\psi \star D^{l_3}\psi \star D^{l_4}\psi \star D^{l_5}N^{2-n}, \\&D^k\left( R^P(D\psi ,\psi )(Ns)^{2-n}\cdot \psi \right) \\&\quad =s^{2-n}\sum _{\sum l_i+\sum {m_j}=k} D^{l_1}R^P\star \underbrace{D^{m_1+1}\phi \star \cdots \star D^{m_{l_1}+1}\phi }_{l_1\text {{-}times}} \star D^{l_2}D\psi \star D^{l_3}\psi \star D^{l_4}\psi \star D^{l_5}N^{2-n}, \\&D^k\left( (s)^{2-n}R^P(\psi ,\psi )D(N)^{2-n}\cdot \psi \right) \\&\quad =s^{2-n}\sum _{\sum l_i+\sum {m_j}=k} D^{l_1}R^P\star \underbrace{D^{m_1+1}\phi \star \cdots \star D^{m_{l_1}+1}\phi }_{l_1\text {{-}times}} \star D^{l_2}\psi \star D^{l_3}\psi \star D^{l_4}\psi \star D^{l_5}DN^{2-n}. \end{aligned}$$

These manipulations allow us to derive the following estimates:

$$\begin{aligned}&\int _\Sigma \left\langle D^k\left( D\left( R^P(\psi ,\psi )(Ns)^{2-n}\right) \partial _i\cdot \psi \right) ,D^k\nabla _t\psi \right\rangle \text { }dV_{g_t} \\&\quad =s^{2-n}\sum _{\sum l_i+\sum {m_j}=k}\int _\Sigma D^{l_1}N^{2-n}\star D^{l_2+1}R^P\star \underbrace{D^{m_1+1}\phi \star \cdots \star D^{m_{l_2+1}+1}\phi }_{(l_2+1)\text {{-}times}}\\&\qquad \star D^{l_3}\psi \star D^{l_4}\psi \star D^{l_5}\psi \star D^k\nabla _t\psi \text { }dV_{g_t} \\&\qquad +s^{2-n}\sum _{\sum l_i+\sum {m_j}=k}\int _\Sigma D^{l_1}N^{2-n}\star D^{l_2}R^P\star \underbrace{D^{m_1+1}\phi \star \cdots \star D^{m_{l_2}+1}\phi }_{l_2\text {{-}times}}\\&\qquad \star D^{l_3}D\psi \star D^{l_4}\psi \star D^{l_5}\psi \star D^k\nabla _t\psi \text { }dV_{g_t} \\&\qquad +s^{2-n}\sum _{\sum l_i+\sum {m_j}=k}\int _\Sigma D^{l_1}D\left( N^{2-n}\right) \star D^{l_2}R^P\star \underbrace{D^{m_1+1}\phi \star \cdots \star D^{m_{l_2}+1}\phi }_{l_2\text {{-}times}}\\&\qquad \star D^{l_3}\psi \star D^{l_4}\psi \star D^{l_5}\psi \star D^k\nabla _t\psi \text { }dV_{g_t} \\&\quad \le C(k)s^{2-n}\left\| N^{2-n}\right\| _{C^k}\left\| R^P\right\| _{C^{k+1}}\sum _{l=0}^k\Vert D\phi \Vert ^{l+1}_{H^r}\Vert \psi \Vert ^3_{H^r}\Vert \nabla _t\psi \Vert _{H^k} \\&\qquad +C(k)s^{2-n}\left\| N^{2-n}\right\| _{C^k}\left\| R^P\right\| _{C^{k}}\sum _{l=0}^k\Vert D\phi \Vert ^{l}_{H^r}\Vert \psi \Vert ^3_{H^r}\Vert \nabla _t\psi \Vert _{H^k} \\&\qquad +C(k)s^{2-n}\left\| N^{2-n}\right\| _{C^{k+1}}\left\| R^P\right\| _{C^{k}}\sum _{l=0}^k\Vert D\phi \Vert ^l_{H^r}\Vert \psi \Vert ^3_{H^r}\Vert \nabla _t\psi \Vert _{H^k}\\&\quad \le C(k)s^{1-n}\left\| N^{2-n}\right\| _{C^{k+1}}\left\| R^P\right\| _{C^{k+1}}\sum _{l=0}^{k+1}F_r(\phi )^{l/2}F_{r-1}(\psi )^2. \end{aligned}$$

To control the last term from the right hand side of (2.5) we calculate

$$\begin{aligned}&D^k\left( (Ns)^{2-n}R^P(\psi ,\psi )R^P(\psi ,\psi )\psi \right) \\&\quad =s^{2-n}\sum _{\sum l_{i_1}+\sum m_{i_2}+\sum q_{i_3}=k} D^{l_1}N^{2-n}\star D^{l_2}R^P\star \underbrace{D^{m_1+1}\phi \star \cdots \star D^{m_{l_2+1}+1}\phi }_{l_2\text {{-}times}}\\&\qquad \star D^{l_3}R^P\star \underbrace{D^{q_1+1}\phi \star \cdots \star D^{q_{l_3+1}+1}\phi }_{l_3\text {{-}times}}\star D^{l_4}\psi \star D^{l_5}\psi \star D^{l_6}\psi \star D^{l_7}\psi \star D^{l_8}\psi . \end{aligned}$$

Consequently, we obtain the following estimate

$$\begin{aligned}&\int _\Sigma \left\langle D^k\left( (Ns)^{2-n}R^P(\psi ,\psi )R^P(\psi ,\psi )\psi \right) ,D^k\nabla _t\psi \right\rangle \text { }dV_{g_t} \\&\quad \le s^{2-n}C(k)\left\| N^{2-n}\right\| _{C^k}\left\| R^P\right\| ^2_{C^k}\sum _{l=0}^{2k}\Vert D\phi \Vert ^{l}_{H^r}\Vert \psi \Vert ^5_{H^r}\Vert \nabla _t\psi \Vert _{H^k}\\&\quad \le s^{1-n}C(k)\left\| N^{2-n}\right\| _{C^k}\left\| R^P\right\| ^2_{C^k}\sum _{l=0}^{2k} F_r(\phi )^{l/2}F_{r-1}(\psi )^3. \end{aligned}$$

The last term of (3.2) can be estimated as

$$\begin{aligned} \int _{\Sigma }\left\langle D^{k}\psi ,e_0\cdot \left( D^*e_0\right) \cdot D^{k+1}\psi \right\rangle \text { }dV_{g_t}\le \Vert D^*e_0\Vert _{L^\infty }\Vert \psi \Vert ^2_{H^r}. \end{aligned}$$

Adding up the different contributions yields the claim. $\square$

#### Remark 3.10

Note that all terms on the right hand side of (3.11) have a similar analytic structure except the terms proportional to $C_3$ and $C_8$, which contain higher powers of the $H^r$-norms of $\phi$ and $\psi$.

### Energy of the coupled pair

Before we prove an energy estimate for the coupled pair, we give more geometric interpretations of the terms appearing in the above estimates. At first, recall that the second fundamental form $\mathrm {I\!I}\in \Gamma (T^*\Sigma ^{\odot 2})$ of a hypersurface $(\left\{ t\right\} \times \Sigma , g_t)\subset (M,h)$ is given by

$$\begin{aligned} \mathrm {I\!I}(X,Y)=\left\langle \nabla _XY-D_XY,\nu \right\rangle _h=\frac{1}{2}\left\langle s^2{\dot{g}}(X,Y)\cdot \partial _t,\nu \right\rangle _h=-\frac{s}{2}{\dot{g}}(X,Y), \end{aligned}$$

where $\nu$ is the future-directed unit normal of the hypersurface.

#### Lemma 3.11

We have the estimates

$$\begin{aligned} \Vert {\dot{g}}\Vert _{C^k}&\le 2s^{-1} \Vert \mathrm {I\!I}\Vert _{C^k},\qquad \Vert De_0\Vert _{L^\infty }\le \Vert \mathrm {I\!I}\Vert _{L^\infty },\qquad \Vert \partial _t\Vert _{C^k}\le s^{-1}\left( 1+\Vert \mathrm {I\!I}\Vert _{C^{k-1}}\right) ,\\ \left\| R^{\Sigma }\right\| _{C^k}&\le C(k)\sum _{l=0}^k\left\| \mathrm {I\!I}\right\| _{C^k}^l\Vert R^M\Vert _{C^k}+C(k)\left\| \mathrm {I\!I}\right\| _{C^k}^2, \\ \left\| R^{SM}\right\| _{C^k}&\le C(k,n)\sum _{l=0}^k\left\| \mathrm {I\!I}\right\| _{C^k}^l\left\| R^M\right\| _{C^k}, \\ \left\| R^{SM}(\partial _t,\cdot )\right\| _{C^k}&\le C(k,n)s^{-1}\sum _{l=0}^{k+1}\left\| \mathrm {I\!I}\right\| ^l_{C^k}\left\| R^M\right\| _{C^k}. \end{aligned}$$

Here, we defined the $C^k$-norm of $R^M$ by taking the *k*th covariant derivative with respect to *h* but taking the norm with respect to the Riemannian reference metric $s^{-2}dt^2+g_t$ in order to get a nonnegative quantity.

#### Proof

The first estimate follows from the definition of $\mathrm {I\!I}$, the second and third from the facts that $\nabla _X\partial _t=\frac{1}{2}{\dot{g}}(X)$ and $e_0=s\partial _t$. Now we recall that for every tensor $T\in \Gamma (T^*\Sigma ^{\otimes k})$ on the manifold *M*, the difference between the covariant derivatives $\nabla$ and *D* can be expressed as

$$\begin{aligned} DT=\nabla T+\mathrm {I\!I}\star T, \end{aligned}$$

which by induction yields

$$\begin{aligned} D^kT=\sum _{l_1+l_2+\sum m_i=k} \underbrace{D^{m_1}\mathrm {I\!I}\star \cdots \star D^{m_{l_1}}\mathrm {I\!I}}_{l_1-\text {times}}\star \nabla ^{l_2}T. \end{aligned}$$

This formula in combination with the Gauß equation

$$\begin{aligned} R^{\Sigma }=R^{M}+\mathrm {I\!I}\star \mathrm {I\!I}\end{aligned}$$

yields the fourth inequality. To prove the last two formulas, we recall that

$$\begin{aligned} R^{SM}(X,Y)\psi =\frac{1}{4}\sum _{\alpha ,\beta =0}^{n-1}R^M\left( X,Y,\partial _{\alpha },\partial _{\beta }\right) \partial _{\alpha }\cdot \partial _{\beta }\cdot \psi \end{aligned}$$

for $X,Y\in \Gamma (TM)$, where $\left\{ \partial _{\alpha }\right\}$ is a local pseudo-orthonormal frame. Therefore, we get in the above situation

$$\begin{aligned} \left( D^k R^{SM}\right) \psi =\sum _{l_1+l_2+\sum m_i=k} \underbrace{D^{m_1}\mathrm {I\!I}\star \cdots \star D^{m_{l_1}}\mathrm {I\!I}}_{l_1-\text {times}}\star \nabla ^{l_2}R^M\star \psi , \end{aligned}$$

which yields the fifth inequality. Similarly, by using the product rule, we obtain

$$\begin{aligned} \left( D^k R^{SM}(\partial _t,\cdot )\right) \psi =\sum _{\sum l_i+\sum m_j=k} D^{l_1}\partial _t\star \underbrace{D^{m_1}\mathrm {I\!I}\star \cdots \star D^{m_{l_2}}\mathrm {I\!I}}_{l_2-\text {times}}\star \nabla ^{l_3}R^M\star \psi \end{aligned}$$

and by using the third inequality we obtain the last one. $\square$

At this point we are ready to control the total energy of $(\phi ,\psi )$, which we define by

$$\begin{aligned} F_r(\phi ,\psi )&=F_r(\phi )+F_{r-1}(\psi )=\sum _{k=0}^rE_k(\phi )+\sum _{k=0}^{r-1}E_k(\psi )+\Vert \psi \Vert _{L^2}^2 \\&=s^2\Vert \partial _t\phi \Vert ^2_{H^r}+\Vert D\phi \Vert ^2_{H^r}+s^2\Vert \nabla _t\psi \Vert ^2_{H^{r-1}}+\Vert D\psi \Vert ^2_{H^{r-1}}+\Vert \psi \Vert _{L^2}^2. \end{aligned}$$

#### Proposition 3.12

Let $T>0$, $r\in \mathbb {N}$, $r>(n-1)/2$ and $(\phi ,\psi )$ be a solution of (2.4), (2.5) such that

$$\begin{aligned}&\phi \in C^0\left( [0,T),H^{r+1}(\Sigma ,P)\right) \cap C^1\left( [0,T),H^r(\Sigma ,P)\right) , \\&\psi \in C^0\left( [0,T),H^{r}\left( M,SM\otimes \phi ^*TP\right) \right) \cap C^1\left( [0,T),H^{r-1}\left( M,SM\otimes \phi ^*TP\right) \right) . \end{aligned}$$

Suppose that ${\dot{s}}\ge 0$ and that the following uniform bounds

$$\begin{aligned}&0<C_2\le N\le C_3,\quad \Vert N\Vert _{C^{r+1}}<C_4,\quad \Vert \nabla _{\nu }N\Vert _{C^{r+1}}<C_5,\\&\Vert \mathrm {I\!I}\Vert _{C^r}<C_6,\quad \left\| R^M\right\| _{C^r}<C_7,\quad \left\| R^P\right\| _{C^{r+1}}<C_8, \quad \Vert \mathrm {I\!I}\Vert _{L^\infty }< C_9s^{-1} \end{aligned}$$

hold for some positive constants $C_i$, $i=1,\ldots ,9$. Suppose finally that there is a uniform bound on all the Sobolev constants of $g_t$. Then the total energy satisfies the following inequality

3.12 $$\begin{aligned} \frac{d}{dt}F_r(\phi ,\psi )\le C\cdot s^{-1}\sum _{l=0}^{2r+4}F_r(\phi ,\psi )^{l/2+1} +C(n-2) {\dot{s}}s^{1-n}\sum _{l=0}^{r}F_r(\phi ,\psi )^{l/2+2}, \end{aligned}$$

where $C>0$ depends on the above bounds.

#### Proof

The estimate is a direct consequence of the evolution inequalities (3.6), (3.10), (3.11), Lemma 3.11 and elementary estimates. $\square$

#### Proof of Theorem 1.1

The existence of a local solution to the system (1.2), (1.3) can be obtained by standard methods, see for example [21, Proposition 9.12]. In order to prove long-time existence, it suffices to prove longtime existence of the solution $(\phi ,\psi )$ of the equivalent system (2.4), (2.5) on the conformal manifold (*M*, *h*).

Moreover, by the continuation criterion for hyperbolic partial differential equations [21, Lemma 9.14] if suffices to obtain a uniform bound on $F_r(\phi ,\psi )$ for all times $t\in [0,\infty )$.

We set $f(t)=(n-2) s^{1-n}{\dot{s}}$. Note that $f(t)$ is integrable with respect to $t$. For $n=2$, this is trivial. For $n>2$, we get due to the assumptions on *s* that

$$\begin{aligned} (n-2)\int _0^\infty \dot{s} s^{1-n}dt=-\int _0^\infty \frac{d}{dt}\left( s^{2-n}\right) dt =-\left( s^{2-n}|_{t=\infty }-s^{2-n}|_{t=0}\right) =s(0)^{2-n}<\infty . \end{aligned}$$

As long as $F_r(\phi ,\psi )\le 1$ we have the differential inequality

$$\begin{aligned} \frac{d}{dt}F_r(\phi ,\psi )\le C\left( s^{-1}(t)+f(t)\right) F_r(\phi ,\psi ), \end{aligned}$$

which can easily be integrated as

$$\begin{aligned} F_r(\phi ,\psi )|_{t=T}\le F_r(\phi ,\psi )|_{t=0}\exp \left( \int _0^T(s^{-1}(t)+f(t))dt\right) . \end{aligned}$$

Now, we set $\Phi :=\int _0^\infty (s^{-1}(t)+f(t))dt<\infty$ and choose $\varepsilon >0$ small enough such that $F_r(\phi ,\psi )|_{t=0}\le (2\Phi )^{-1}$. Suppose that $T_0$ is the first time for which $F_r(\phi ,\psi )|_{T_0}=1$. However, the energy inequality from above gives

$$\begin{aligned} F_r(\phi ,\psi )|_{t=T_0}\le \Phi F_r(\phi ,\psi )|_{t=0}=\frac{1}{2}<1, \end{aligned}$$

which yields a contradiction. Therefore we can conclude that

$$\begin{aligned} F_r(\phi ,\psi )<1<\infty \end{aligned}$$

for all times $t\in [0,\infty )$ completing the proof. $\square$

#### Proof of Theorem 1.3

The proof is as above but we additionally assume that $\psi \equiv 0$. In this case, we just need the assumptions in Proposition 3.7 and not the slightly stronger ones in Proposition 3.8. As the spinor is not involved, we obviously can also remove the spin condition. $\square$
